# Fewer Functional Deficits and Reduced Cell Death after Ranibizumab Treatment in a Retinal Ischemia Model

**DOI:** 10.3390/ijms19061636

**Published:** 2018-05-31

**Authors:** Marina Palmhof, Stephanie Lohmann, Dustin Schulte, Gesa Stute, Natalie Wagner, H. Burkhard Dick, Stephanie C. Joachim

**Affiliations:** Experimental Eye Research, University Eye Hospital, Ruhr-University Bochum, In der Schornau 23-25, 44892 Bochum, Germany; marina.palmhof@rub.de (M.P.); stephanie.lohmann@rub.de (S.L.); dustin-schulte@web.de (D.S.); gesa.stute@rub.de (G.S.); natalie.wagner@rub.de (N.W.); Burkhard.Dick@kk-bochum.de (H.B.D.)

**Keywords:** ischemia/reperfusion, retina, VEGF, bevacizumab, ranibizumab, ERG, retinal ganglion cell, apoptosis, autophagocytosis

## Abstract

Retinal ischemia is an important factor in several eye disorders. To investigate the impact of VEGF inhibitors, as a therapeutic option, we studied these in a retinal ischemia animal model. Therefore, animals received bevacizumab or ranibizumab intravitreally one day after ischemia induction. Via electroretinography, a significant decrease in a- and b-wave amplitudes was detected fourteen days after ischemia, but they were reduced to a lesser extent in the ranibizumab group. Ischemic and bevacizumab retinae displayed fewer retinal ganglion cells (RGCs), while no significant cell loss was noted in the ranibizumab group. Apoptosis was reduced after therapy. More autophagocytotic cells were observed in ischemic and bevacizumab eyes, but not in ranibizumab eyes. Additionally, more microglia, as well as active ones, were revealed in all ischemic groups, but the increase was less prominent under ranibizumab treatment. Fewer cone bipolar cells were detected in ischemic eyes, in contrast to bevacizumab and ranibizumab-treated ones. Our results demonstrate a reduced apoptosis and autophagocytosis rate after ranibizumab treatment. Furthermore, a certain protection was seen regarding functionality, RGC, and bipolar cell availability, as well as microglia activation by ranibizumab treatment after ischemic damage. Thus, ranibizumab could be an option for treatment of retinal ischemic injury.

## 1. Introduction

Retinal diseases, like age-related macular degeneration (AMD), diabetic retinopathy, glaucoma, and retinal vein occlusion, are leading causes of blindness worldwide [1]. These retinal pathologies are associated with ischemic processes, which represent one important cause for visual impairment throughout disease development [2,3,4]. During retinal ischemia the blood flow is reduced, which leads to an under-supply of oxygen and other nutrients in the different retinal layers [2,5,6]. This process is followed by a restoration of the retinal blood flow, which results in increased oxidative stress and subsequent inflammatory responses in the tissue [7]. Consequently, morphological and functional changes of different retinal cell types occur, as shown by several studies [5,6,8,9,10,11]. The basis of these studies is the retinal ischemia/reperfusion animal model. By increasing the intraocular pressure (IOP) for a definite period such retinal circulatory disorders can be induced. The high IOP leads to a compression of retinal blood vessels which, in turn, leads to an impaired blood flow. Additionally, oxidative stress, and in this context concentrations of reactive oxygen species, are increased by the subsequent natural reperfusion, which additionally leads to massive tissue damage and inflammation [8,12,13]. Our research group, as well as others, showed cell loss and damage of various retinal cell types in this animal model [14,15]. Especially, retinal ganglion cells (RGCs) and amacrine cells are known to be affected. In addition to a detected inflammatory response by microglia after ischemia/reperfusion, a functional loss of inner retinal cell layers could also be measured by full-field flash electroretinography [11].

A current therapeutic approach to the above-mentioned degenerative retinopathies is the use of intravitreous vascular endothelial growth factor (VEGF) inhibitors, like bevacizumab (Avastin, Genentech) and ranibizumab (Lucentis, Novartis), in patients [16]. Bevacizumab is a full-length humanized monoclonal antibody, while ranibizumab is a humanized recombinant monoclonal antibody fragment (Fab) and just a third of the size of bevacizumab. Bevacizumab is directed against all isoforms of VEGF, whereas ranibizumab specifically binds to all VEGF-A isoforms. The Food and Drug Administration (FDA) has approved bevacizumab as first-line treatment for human metastatic colorectal cancer for intravenous use [17]. However, it is also used as an off-label drug for ocular diseases, including AMD and diabetic macular edema, since 2005 [18]. In contrast, ranibizumab has been FDA approved for intravitreous injections for the treatment of AMD, diabetic retinopathy, retinal vein occlusion, and diabetic macular edema since 2006 [16,19]. In its function to inhibit the VEGF-induced endothelial cell proliferation, ranibizumab is 10–20-fold more potent. Another substance, Aflibercept (Eylea, Bayer), a recombinant fusion protein, has been approved for treatment of several vascular retinal diseases. The highest potential against retinal vascular diseases among these three anti-VEGF drugs is still part of the controversial clinical discussion.

The goal of our study was to evaluate the effects of bevacizumab and ranibizumab treatment on retinal cell types after ischemia/reperfusion injury in an animal model. Some clinical trials suggest that both inhibitors, ranibizumab and the off-label used bevacizumab, showed similar efficacy. Thus, we wanted to investigate and compare their impact on a cellular and molecular level. To this end, we analyzed ischemic retinae after one single dose of the two intravitreously administered anti-VEGF drugs using electroretinography, immunohistology, and quantitative real-time PCR (qRT-PCR). The VEGF inhibitors were injected in a dose of 0.05 mg per 5 µL one day after ischemia induction. Neuronal activity, different retinal cell types such as RGCs and microglia, various cell death mechanisms, including apoptosis, as well as autophagy, were investigated. All analyses were performed fourteen days after induction of ischemia/reperfusion.

## 2. Results

The comparison of the two VEGF inhibitors bevacizumab and ranibizumab was the main aspect of our study. The findings should contribute to a better understanding of the mode of action of the two different inhibitors. Thus, we had three ischemic groups for the investigations, one ischemia only and two which additionally received anti-VEGF treatment one day later. These groups were compared to a control group fourteen days after ischemia induction.

**Figure 1:**
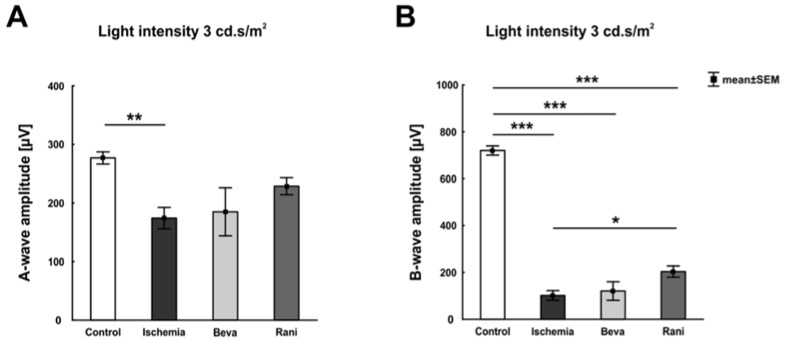
ERG measurements of all groups, control, untreated ischemic, and the VEGF treated ones (beva and rani), were performed. The recording at a light intensity of 3 cd·s/m^2^ is pictured. (**A**) A significant decrease of the a-wave amplitude was observed in the ischemic group (*p* = 0.007) compared to control. The bevacizumab- (*p* = 0.06) and ranibizumab-treated groups (*p* = 0.32) showed a lesser reduced amplitude at this light intensity, when compared to control eyes; (**B**) Additionally, the b-wave amplitude was lower in all ischemic eyes, the untreated (*p* < 0.001) and the anti-VEGF treated ones (*p* < 0.001), in comparison to the control group. The b-wave amplitude of the ranibizumab-treated group was less diminished as well. A significant increase of the amplitude could be detected here in comparison to the ischemic group (*p* = 0.03). *: *p* < 0.05; **: *p* < 0.01; ***: *p* < 0.001. Abbreviations: Beva: bevacizumab, Rani: ranibizumab. *n* = 5–6/group.

The electroretinogram (ERG) measurements showed a significant, light intensity-dependent, decrease of the a-wave amplitude in all ischemic groups (*p* < 0.05; Supplement Table S1A) in comparison to control eyes. Interestingly, this amplitude reduction was not as prominent in the ranibizumab group, where a significant amplitude decrease was only measured at 0.3 candela (Figure 1A; Supplement Table S1A). Regarding the b-wave, a lower amplitude could also be revealed in all ischemic eyes (*p* < 0.001; Supplement Table S1B). However, the b-wave amplitude of ranibizumab-treated eyes was less decreased. With a light intensity of 3 candela, the amplitude was in fact significantly higher than the one of ischemic eyes (*p* = 0.03; Figure 1B; Supplement Table S1B).

**Figure 2:**
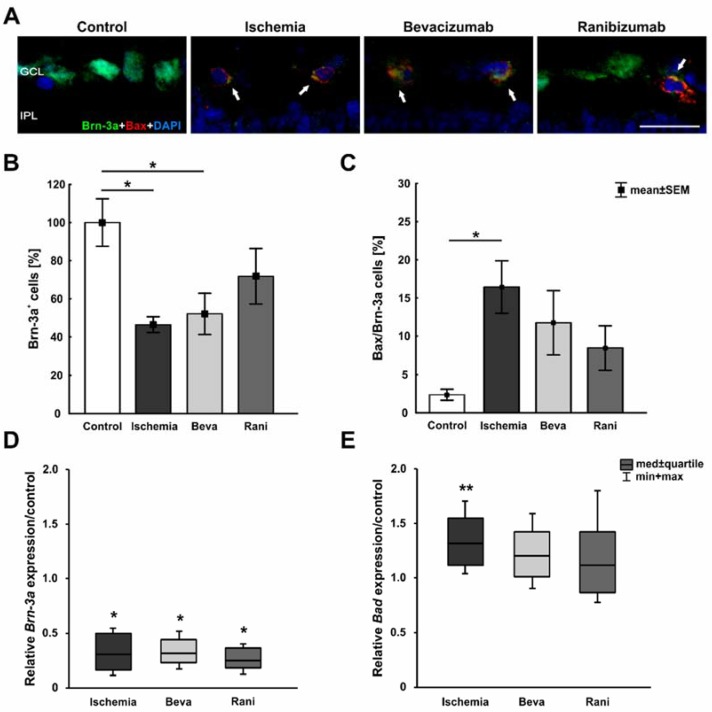

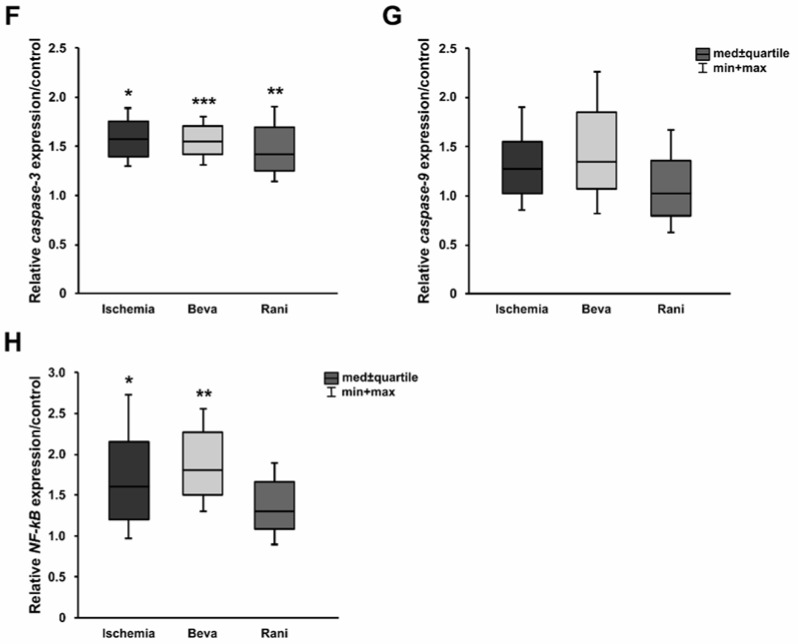
(**A**) Exemplary retinal cross-sections of the four groups stained with anti-Brn-3a for RGCs (green), Bax for apoptotic cells (red), and DAPI for cell nuclei (blue). Fewer Brn-3a^+^ RGCs and more Bax^+^ apoptotic cells were noted in ischemic retinae; (**B**) A significant loss of Brn-3a^+^ ganglion cells was revealed in the ischemic (*p* = 0.02) and the bevacizumab group (*p* = 0.04), but not in ranibizumab-treated retinae (*p* = 0.35); (**C**) Significantly more apoptotic cells could be observed in ischemic eyes (*p* = 0.03), but not in the groups treated with bevacizumab (*p* = 0.22) and ranibizumab (*p* = 0.57); (**D**) A significant reduction of relative *Brn-3a (Pou4f1)* mRNA expression was measured in all ischemic groups via qRT-PCR. The untreated (*p* = 0.023), the bevacizumab- (*p* = 0.018), and ranibizumab-treated eyes (*p* = 0.017) expressed a lower *Brn-3a (Pou4f1)* mRNA level than the controls; (**E**) The relative *Bad* mRNA expression was significantly elevated in ischemic (*p* = 0.002) eyes. No effect in *Bad* mRNA expression could be detected between the control and the bevacizumab- (*p* = 0.131) and ranibizumab-treated groups (*p* = 0.491); (**F**) Regarding the relative *caspase-3* mRNA expression, a significant up-regulation was noted in ischemic (*p* = 0.026), bevacizumab (*p* < 0.001), and ranibizumab-treated retinae (*p* = 0.009), when compared to the control group; (**G**) Analysis of relative *caspase-9* mRNA expression revealed no differences in the expression level between the control and all ischemic eyes (ischemia: *p* = 0.131, beva: *p* = 0.118, rani: *p* = 0.858); (**H**) A significant elevation of *NF-κB* mRNA levels was detected in the ischemic (*p* = 0.034) and bevacizumab-treated group (*p* = 0.008) in comparison to control retinae. No differences in relative *NF-κB* mRNA expression were seen between the control and ranibizumab-treated retinae (*p* = 0.069). *: *p* < 0.05; **: *p* < 0.01; ***: *p* < 0.001. Abbreviations: GCL: ganglion cell layer, IPL: inner plexiform layer, Beva: bevacizumab, Rani: ranibizumab. Arrows: colocalization. Scale bar: 20 µm. Immunohistology: *n* = 5–6/group; qRT-PCR: *n* = 4/group.

RGCs were stained with the specific marker anti-Brn-3a [20] and, additionally, apoptotic cells were marked using anti-Bax. Fourteen days after ischemia induction, fewer Brn-3a^+^ RGCs could be observed in ischemic and bevacizumab-treated eyes, while ranibizumab-treated retinae looked like those of the control group. More Bax^+^ apoptotic cells were seen in all ischemic eyes, with fewer Bax^+^ cells in the ranibizumab group (Figure 2A). The positive signals were located in the GCL (Supplement Figure S1A). Quantification of the immunohistological staining confirmed this impression. Compared to controls (100% ± 12.5%), significantly fewer Brn-3a^+^ RGCs were detected in the ischemic (46.5% ± 4.1%; *p* = 0.02) and bevacizumab groups (52.1% ± 10.8%; *p* = 0.04), but not in the ranibizumab group (71.8% ± 14.5%; *p* = 0.35; Figure 2B). A significant increase of apoptotic cells could be noted in ischemic eyes (16.4 ± 3.4%; *p* = 0.03) in comparison to controls (2.4% ± 0.7%), but not in bevacizumab (11.8% ± 4.2%; *p* = 0.22) and ranibizumab (8.5% ± 2.9%; *p* = 0.57) treated eyes (Figure 2C). There was no difference between ischemic retinae and those treated with the VEGF inhibitors (beva: *p* = 0.75; rani: *p* = 0.27), although a slight trend to fewer Bax^+^ cells was observed in the ranibizumab-treated group in relation to the ischemic group (*p* = 0.27).

In order to evaluate the *Brn-3a* (*Pou4f1*; retinal ganglion cells), *Bad* (pro-apoptotic protein of the intrinsic apoptotic pathway), *caspase-3*, *caspase-9*, and *NF-κB* (proteins of the extrinsic apoptotic pathway) expression on mRNA level, qRT-PCR analyses were performed. Compared to controls, a significant down-regulation of relative *Brn-3a* expression was detected in all ischemic groups, the untreated (0.3-fold expression; *p* = 0.023) as well as the bevacizumab (0.312-fold expression; *p* = 0.018) and ranibizumab-treated eyes (0.251-fold expression; *p* = 0.017; Figure 2D). Regarding the relative *Bad* expression, a significant up-regulation could be measured in ischemic (1.32-fold expression; *p* = 0.002) retinae. No differences in *Bad* mRNA levels could be noted between the control and the bevacizumab- (1.198-fold expression; *p* = 0.131) and the ranibizumab-treated groups (1.117-fold expression; *p* = 0.491; Figure 2E). Investigation of the relative *caspase-3* mRNA expression revealed a significant elevation in ischemic (1.579-fold expression; *p* = 0.026) as well as bevacizumab- (1.556-fold expression; *p* < 0.001) and ranibizumab-treated retinae (1.42-fold expression; *p* = 0.009; Figure 2F) in relation to the control group. No differences could be noted regarding *caspase-9* mRNA levels. The relative expression of *caspase-9* mRNA in all three ischemic groups, the untreated (1.28-fold expression; *p* = 0.131), the bevacizumab-treated (1.35-fold expression; *p* = 0.118), and the ranibizumab-treated (1.027-fold expression; *p* = 0.858; Figure 2G), was similar to the control group. However, a significant increase of relative *NF-κB* mRNA levels could be detected in ischemic (1.606-fold expression; *p* = 0.034) and bevacizumab-treated retinae (1.803-fold expression; *p* = 0.008). No difference was noted between the control and ranibizumab-treated group (1.303-fold expression; *p* = 0.069; Figure 2H).

**Figure 3:**
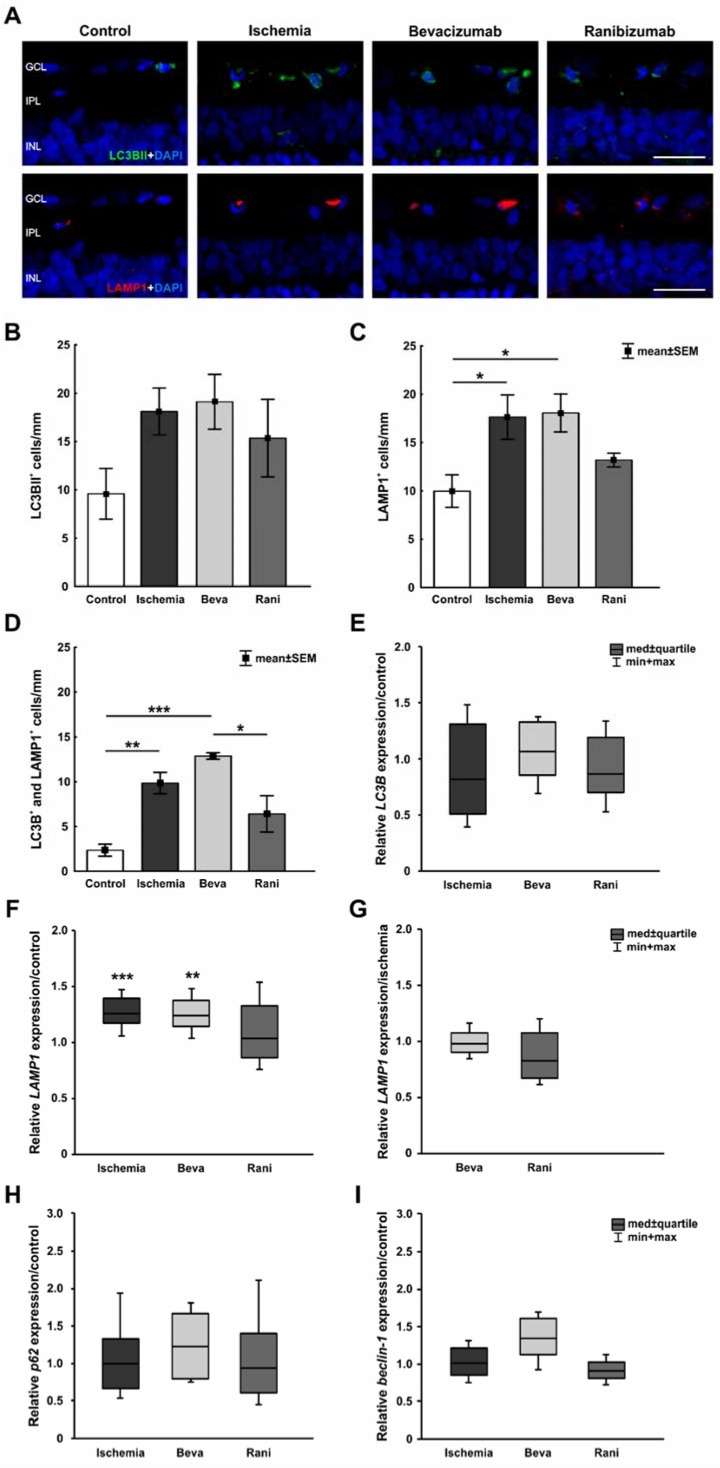
(**A**) Early autophagy was labelled with anti-LC3BII (green), while anti-LAMP1 was used to visualize the late autophagy (red) and DAPI-marked cell nuclei (blue). More autophagocytotic cells were noted after ischemia induction; (**B**) LC3BII^+^ cell counts revealed no differences between all ischemic groups (*p* > 0.05), when compared to controls, although the number of positive cells was a little bit higher in those groups; (**C**) Regarding LAMP1 staining, significantly more LAMP1^+^ autophagocytotic cells were detected in ischemic (*p* = 0.04) and bevacizumab-treated (*p* = 0.03) eyes, when compared to controls. The retinae treated with ranibizumab displayed fewer LAMP1^+^ cells, no significant difference could be observed in relation to control eyes (*p* = 0.6); (**D**) Equally, evaluation of colocalized LC3BII^+^ and LAMP1^+^ cells revealed significantly increased LC3BII^+^ and LAMP1^+^ cell numbers in the ischemic (*p* = 0.007) and bevacizumab-treated groups (*p* < 0.001), but not in ranibizumab-treated retinae (*p* = 0.2); (**E**) Via qRT-PCR no differences in relative *LC3B* mRNA expression were noted when compared the untreated (*p* = 0.46) and treated ischemic groups (beva: *p* = 0.625; rani: *p* = 0.38) to the control group; (**F**) A significant up-regulation of *LAMP1* mRNA levels could be measured in the untreated ischemic (*p* < 0.001) and bevacizumab-treated groups (*p* = 0.007) in relation to control retinae. No differences in relative *LAMP1* mRNA expression were seen between the control and ranibizumab-treated retinae (*p* = 0.769); (**G**) In comparison to the ischemic group, a slight trend to less LAMP1 mRNA expression was noted in retinae treated with ranibizumab (*p* = 0.13); (**H**) Regarding *p62* mRNA expression, no effect on the expression level was observed between control retinae and all ischemic groups (*p* > 0.05); (**I**) The relative *beclin-1* mRNA expression levels in the ischemic (*p* = 0.888), bevacizumab (*p* = 0.086), and ranibizumab-treated eyes (*p* = 0.178) were similar to the control group. *: *p* < 0.05; **: *p* < 0.001; ***: *p* < 0.001. Abbreviations: GCL: ganglion cell layer, IPL: inner plexiform layer, INL: inner nuclear layer, Beva: bevacizumab, Rani: ranibizumab. Scale bar: 20 µm. Immunohistology: *n* = 5–6/group; qRT-PCR: *n* = 4/group.

A specific antibody against LC3BII was used to visualize early autophagocytotic processes. Late autophagy was detected using anti-LAMP1. More LC3BII^+^, as well as LAMP1^+^ cells, were observed in ischemic eyes and those with bevacizumab treatment. In comparison, the ranibizumab-treated retinae seemed to reveal less autophagocytotic cells (Figure 3A). These were mainly present in the GCL (Supplement Figure S1B). Statistically, no differences could be seen in the number of LC3BII^+^ cells between the control (9.6 ± 2.6 cells/mm) and ischemic group (18.1 ± 2.4 cells/mm; *p* = 0.29). Additionally, a comparison of the control group with the bevacizumab (19.1 ± 2.8 cells/mm; *p* = 0.20) and the ranibizumab group (15.4 ± 4 cell/mm; *p* = 0.61) revealed no differences. Although, a trend to more LC3BII^+^ cells could be noted in all ischemic groups compared to control retinae (Figure 3B). Regarding late autophagocytotic processes, ischemic (17.6 ± 2.3 cells/mm; *p* = 0.04) and bevacizumab-treated (18.1 ± 2 cells/mm; *p* = 0.03) eyes displayed significantly more LAMP1^+^ autophagocytotic cells than the ranibizumab group (13.2 ± 0.7 cells/mm; *p* = 0.6), when compared to controls (10 ± 1.7 cells/mm; Figure 3C). Furthermore, colocalization of LC3BII^+^ and LAMP1^+^ cells was evaluated. In comparison to the control group (2.4 ± 0.7 cells/mm), a significant increase of colocalized LC3BII^+^ and LAMP1^+^ cells was noted in the ischemic (9.9 ± 1.2 cells/mm; *p* = 0.007) and bevacizumab-treated groups (12.9 ± 0.4 cells/mm; *p* < 0.001), but not in eyes which received treatment with ranibizumab (6.4 ± 2.0; *p* = 0.2; Figure 3D). However, a significant decrease of LC3BII^+^ and LAMP1^+^ cells was detected in ranibizumab-treated eyes in comparison to bevacizumab-treated ones (*p* = 0.02). Comparison of anti-VEGF treated retinae with the ischemic group revealed no differences (beva: *p* = 0.4; rani: *p* = 0.3).

Additionally, *LC3B* mRNA expression was analyzed via qRT-PCR. Here, no differences in expression levels were seen comparing the control group and ischemic (0.821-fold expression; *p* = 0.46), bevacizumab- (1.069-fold expression; *p* = 0.625), and ranibizumab-treated retinae (0.872-fold expression; *p* = 0.38; Figure 3E). Indeed, a significant increase in *LAMP1* mRNA levels could be detected in the ischemic (1.258-fold expression; *p* < 0.001) and bevacizumab groups (1.235-fold expression; *p* = 0.007) in relation to control retinae. In accordance with the immunohistological data, there were no differences in *LAMP1* mRNA expression between control and ranibizumab-treated eyes (1.038-fold expression; *p* = 0.769; Figure 3F). Furthermore, a slight trend to less *LAMP1* expression was noted in the ranibizumab group (0.825-fold expression; *p* = 0.13), when compared to the ischemic group (Figure 3G). To take a closer look at the activation of autophagy, relative expression of *p62* and *beclin-1* mRNA were analyzed. However, no effects could be seen in the expression levels in all ischemic groups. Regarding relative *p62* expression, the untreated (0.999-fold expression; *p* = 0.968), the bevacizumab- (1.233-fold expression; *p* = 0.314), and the ranibizumab-treated retinae (0.942-fold expression; *p* = 0.748) displayed a similar *p62* level in comparison to the control group (Figure 3H). Equally, no differences in *beclin-1* mRNA expression could be detected between the control group and all ischemic retinae, without (1.008-fold expression; *p* = 0.888) and with treatment (beva: 1.339-fold expression; *p* = 0.086, rani: 0.905-fold expression; *p* = 0.178; Figure 3I).

**Figure 4:**
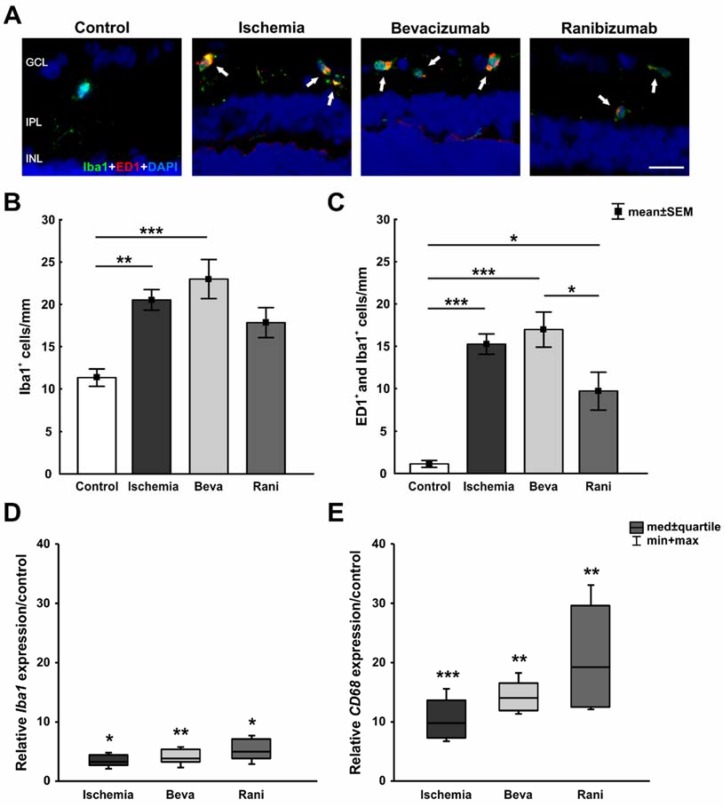
(**A**) Microglia were marked with an anti-Iba1 antibody (green), while activated microglia were stained with anti-ED1 (red). After ischemia induction more Iba1^+^ and ED1^+^ microglia were observed; (**B**) Counts displayed a significant increase of Iba1^+^ microglia in the ischemic (*p* = 0.007) and the bevacizumab (*p* < 0.001) groups, when compared to controls. A trend of fewer Iba1^+^ cells was noted in eyes treated with ranibizumab (*p* = 0.07) in comparison to control eyes; (**C**) All ischemic retinae displayed significantly more activated microglia (ischemia: *p* < 0.001, beva: *p* < 0.001, rani: *p* = 0.01), compared to the control group. A significant decrease of ED1^+^ as well as Iba1^+^ cells was visible in ranibizumab-treated retinae compared to bevacizumab-treated ones (*p* = 0.04); (**D**) On mRNA level, a significant up-regulation of *Iba1* mRNA expression could be shown in all ischemic groups, the untreated (*p* = 0.01), the bevacizumab-treated (*p* = 0.004) as well as the ranibizumab-treated ones (*p* = 0.014), in relation to controls; (**E**) Additionally, a significant elevation of relative *CD68* mRNA (activated microglia) expression was measured in these groups (ischemia: *p* < 0.001, beva: *p* = 0.001, rani: *p* = 0.001) compared to control retinae. *: *p* < 0.05; **: *p* < 0.001; ***: *p* < 0.001. Abbreviations: GCL: ganglion cell layer, IPL: inner plexiform layer, INL: inner nuclear layer, Beva: bevacizumab, Rani: ranibizumab. Arrows: colocalization. Scale bar: 20 µm. Immunohistology: *n* = 5–6/group; qRT-PCR: *n* = 4/group.

The anti-Iba1 antibody was used to detect the microglia population, while activated microglia were additionally marked with anti-ED1. More Iba1^+^ and ED1^+^ microglia were seen after ischemia induction, while staining intensity in ranibizumab-treated retinae seemed to be less prominent (Figure 4A). Microglia and their activated type were observed to be distributed in the GCL, IPL, and INL (Supplement Figure S1C). Statistical analyses displayed a significant increase of Iba1^+^ microglia in the ischemic (20.5 ± 1.2 cells/mm; *p* = 0.007) and the bevacizumab (23 ± 2.3 cells/mm; *p* < 0.001) group in comparison to the control group (11.3 ± 1 cells/mm). A trend to less Iba1^+^ cells was revealed in ranibizumab-treated retinae (17.8 ± 1.8 cells/mm; *p* = 0.07), when compared to controls (Figure 4B). No significant differences were detected comparing the groups with anti-VEGF treatment (beva: *p* = 0.75; rani: *p* = 0.63) and the ischemic group (Figure 4B). Also, a significant microglia activation could be noted in all ischemic retinae (ischemia: 15.3 ± 1.2 cells/mm; *p* < 0.001, beva: 17 ± 2.1 cells/mm; *p* < 0.001, rani: 9.7 ± 2.2 cells/mm; *p* = 0.01), in contrast to the control group (1.1 ± 0.4 cells/mm; Figure 4C). However, a significant decrease of ED1^+^ cells was detected in ranibizumab-treated eyes in comparison to bevacizumab-treated ones (*p* = 0.04). Furthermore, a trend to fewer activated microglia could be observed in ranibizumab-treated retinae in relation to the ischemic group (*p* = 0.11).

Regarding the relative *Iba1* expression, a significant up-regulation in the mRNA level was measured in all ischemic groups. The untreated (3.214-fold expression; *p* = 0.01), the bevacizumab-treated (3.866-fold expression; *p* = 0.004), as well as the ranibizumab-treated retinae (4.877-fold expression; *p* = 0.014) displayed a higher *Iba1* level in relation to controls (Figure 4D). Equally, *CD68* mRNA (activated microglia) expression was significantly increased in these groups (ischemia: 9.714-fold expression; *p* < 0.001, beva: 13.971-fold expression; *p* = 0.001, rani: 19.218; *p* = 0.001) compared to the control retinae (Figure 4E).

**Figure 5:**
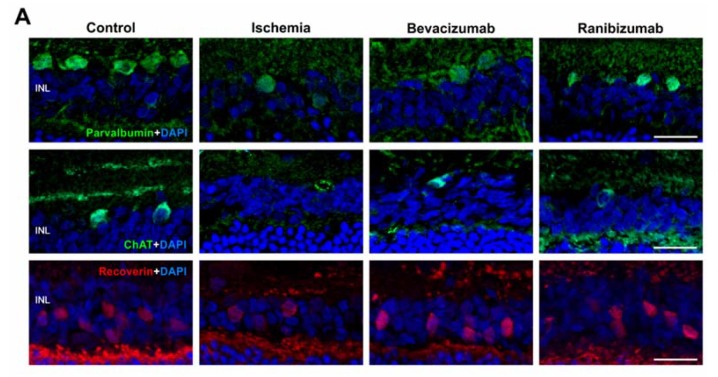

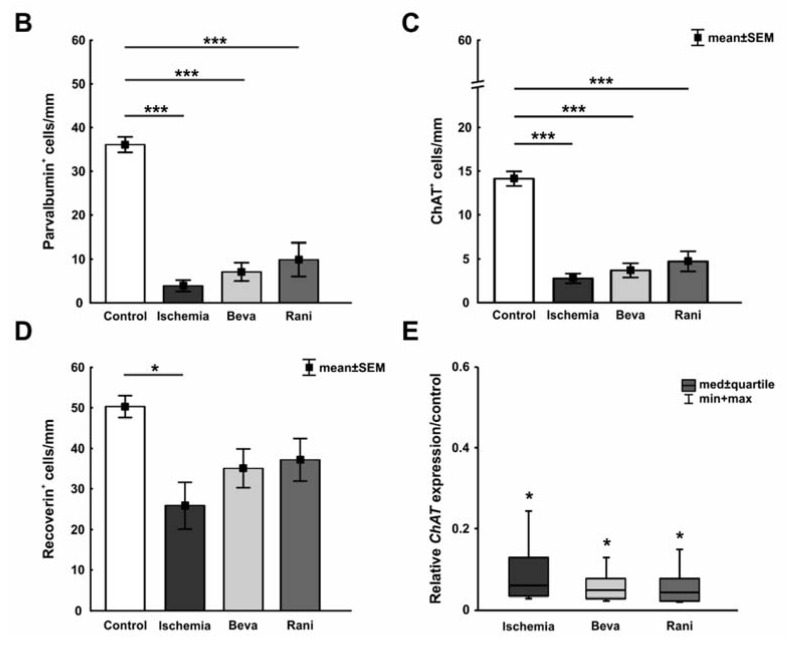
(**A**) Anti-parvalbumin was used to stain AII amacrine cells (green) and anti-ChAT to mark cholinergic amacrine cells (green), while cone bipolar cells were labelled with anti-recoverin (red). Cell nuclei were visualized with DAPI (blue). In comparison to the control group, fewer amacrine cells, as well as cone bipolar cells, were visualized after ischemic injury. The loss of bipolar cells seemed to be less prominent after bevacizumab and ranibizumab treatment; (**B**) Significantly fewer amacrine cells were counted in ischemic eyes (*p* < 0.001) and after treatment with bevacizumab (*p* < 0.001) and ranibizumab (*p* < 0.001) compared to the control group; (**C**) Regarding cholinergic amacrine cells, in contrast to control eyes a significant loss of ChAT^+^ cells was revealed in the ischemic (*p* < 0.001), in the bevacizumab (*p* < 0.001), and ranibizumab-treated group (*p* < 0.001); (**D**) A significant loss of cone bipolar cells could be detected in the ischemic group (*p* = 0.01), but not in bevacizumab- (*p* = 0.17) and ranibizumab-treated eyes (*p* = 0.28) in relation to the control retinae; (**E**) Via qRT-PCR, a significant down-regulation of relative *ChAT* mRNA expression could be shown in the untreated (*p* = 0.032), the bevacizumab-treated (*p* = 0.015), and the ranibizumab-treated (*p* = 0.018) ischemic groups, when compared to control eyes. *: *p* < 0.05; ***: *p* < 0.001. Abbreviations: INL: inner nuclear layer, Beva: bevacizumab, Rani: ranibizumab. Scale bar: 20 µm. Immunohistology: *n* = 5–6/group; qRT-PCR: *n* = 4/group.

AII amacrine cells were detected with the marker anti-parvalbumin, cholinergic amacrine cells with anti-ChAT, and cone bipolar cells were stained using anti-recoverin. Fewer parvalbumin^+^ as well as ChAT^+^ amacrine cells could be revealed in all groups after ischemia, when compared to controls. The number of cone bipolar cells was also reduced in all ischemic groups, which seemed to be less decreased after bevacizumab and ranibizumab treatment (Figure 5A). The cell bodies of all three cell types were noted in the INL. Furthermore, some displaced cholinergic amacrine cells were localized in the GCL. Regarding ChAT immunoreactivity, a distinct stratification in the IPL was observed in the control group and a weakened one in the ranibizumab-treated group, while it was not present after ischemia induction and bevacizumab treatment. Additionally, immunolabeling of parvalbumin was noted in the IPL of control retinae, but not in the ischemic and anti-VEGF treated groups. No recoverin immunolabeling was detected in the IPL of all groups (Supplement Figure S1D). This observation was confirmed by statistical analyses. All ischemic groups, the untreated (3.9 ± 1.3 cells/mm; *p* < 0.001), as well as the bevacizumab- (7.1 ± 2.1 cells/mm; *p* < 0.001) and ranibizumab-treated eyes (9.9 ± 3.8 cells/mm; *p* < 0.001), displayed a significant lower number of parvalbumin^+^ amacrine cells in relation to the controls (36.1 ± 1.8 cells/mm; Figure 5B). Equally, a significant loss of ChAT^+^ amacrine cells was detected in untreated ischemic (2.8 ± 0.6 cells/mm; *p* < 0.001) as well as bevacizumab- (3.7 ± 0.8 cells/mm; *p* < 0.001) and ranibizumab-treated retinae (4.7 ± 1.2 cells/mm; *p* < 0.001) in comparison to the control eyes (14.1 ± 0.8 cells/mm; Figure 5C). Significantly fewer recoverin^+^ cone bipolar cells were observed in the retinae of the ischemic group (25.9 ± 5.7 cells/mm; *p* = 0.01), but not in the eyes treated with bevacizumab (35.1 ± 4.8 cells/mm; *p* = 0.17) and ranibizumab (37.2 ± 5.3 cells/mm; *p* = 0.28), when compared to the control group (50.3 ± 2.7 cells/mm; Figure 5D). No significant differences could be noted between retinae treated with the VEGF inhibitors (beva: *p* = 0.57; rani: *p* = 0.4) and ischemic eyes.

qRT-PCR analyses were performed to investigate the *ChAT* expression on mRNA level. A significant down-regulation of relative *ChAT* expression was measured in all ischemic groups, the untreated (0.059-fold expression; *p* = 0.032), as well as the bevacizumab (0.047-fold expression; *p* = 0.015) and ranibizumab-treated (0.041-fold expression; *p* = 0.018; Figure 5E) in comparison to the control retinae.

## 3. Discussion

The focus of our study was the investigation of an intravitreal anti-VEGF treatment after retinal ischemic injury, including the comparison of two different anti-VEGF drugs. Therefore, we used two VEGF inhibitors, bevacizumab and ranibizumab, which were applied one day after ischemia induction. All analyses were performed fourteen days after induction of ischemia/reperfusion. Overall, we found a better protection by ranibizumab. Especially a reduced autophagy and apoptosis rate was detected after ranibizumab treatment, when we compared all ischemic groups to the control group. There were no significant differences between the untreated ischemic and the anti-VEGF treated groups. However, in comparison to ischemic retinae, a trend to fewer apoptotic and autophagocytotic cells was observed in ranibizumab-treated eyes.

Various neurodegenerative eye diseases, including AMD, diabetic retinopathy, glaucoma, and retinal vascular occlusion, are multifactorial. Consequently, the pathology and the exact causes of these diseases are still unclear. However, it is known that they are accompanied by ischemic processes [1]. Due to the complexity, there is still no optimal treatment option available. A therapeutic measure is currently the use of VEGF inhibitors, like bevacizumab and ranibizumab. In this context, the neurodegenerative, as well as neuroprotective, impact of VEGF is still part of the discussion. Previous studies show that retinal ischemic events stimulate the secretion of VEGF and that the increased expression triggers vascular angiogenesis and vascular permeability in many retinopathies [21,22,23]. Additionally, in clinical trials patients with neovascular AMD, diabetic macular edema, or retinal vein occlusion had an increased VEGF expression in the vitreous or aqueous humor [24,25,26,27]. It is still a matter of discussion which of the two inhibitors, the full-length antibody (bevacizumab) or the antibody fragment (ranibizumab), shows greater effectiveness and, therefore, leads to better treatment results. The toxicity of the VEGF inhibitors has already been investigated in vivo and in vitro [28,29]. Thaler et al. performed electron microscopy and evaluated retrogradely-labelled RGCs after intravitreal injection of bevacizumab and ranibizumab in healthy rats and those with NMDA-induced RGC damage. Neither in healthy animals, nor in NMDA treated animals, were any differences in structure, morphology, nor the number of RGCs after anti-VEGF treatment found in comparison to the respective control groups [28].

However, the role of VEGF in the nervous system is not only limited to the regulation and development of vessel growth, it also acts directly on different retinal neurons and causes damage [30,31]. Certainly, the impact on specific retinal cells has been only marginally analyzed. It is known, that in human retina VEGF is expressed by all major classes of neurons. Especially in cell bodies of RGCs and amacrine cells, VEGF was detected [32]. Additionally, in the rat retina, immunoreactivity of VEGF could be proven in the GCL and INL. Here, a predominant localization of VEGF is assumed in neurons, while it is scarcely expressed in retinal vessels [33]. Therefore, we examined the effect of the two inhibitors after ischemia in a retinal ischemia/reperfusion animal model via electroretinography, immunohistology, and qRT-PCR.

### 3.1. Certain Preservation of the Neuronal Function

Comparative clinical trials for the treatment of neovascular AMD showed that the effects of bevacizumab and ranibizumab on visual acuity are equivalent [34,35]. Therefore, it was of great interest to measure neuronal activity under the impact of anti-VEGF treatment after ischemia induction in an animal model via electroretinography. The reduction of the a- and b-wave amplitudes after ischemia could be demonstrated in various studies [2,11]. Specific investigation of retinal function has only been implemented in a few animal studies, which noted a reduced retinal function. However, in these an inhibition or neutralization of VEGF without a prior retinal injury was made [36,37]. In our study, the bevacizumab-treated group was comparable with the ischemic group regarding the level of a- and b-wave amplitudes, which reflect the activity of the photoreceptors and the cell activity in the inner nuclear layer (INL), respectively. Interestingly, a tendentially higher a- and b-wave amplitude was measured in ranibizumab-treated in comparison to ischemic eyes. This allows us to assume that a certain preservation of the function of photoreceptors and neurons in the INL occurred. These findings are in concordance with a previous study of our group, which analyzed the effect of a later ranibizumab application 21 days after ischemia. There, a protection of the rhodopsin^+^ area was observed after ranibizumab treatment compared to ischemic retinae [38].

### 3.2. Some Prevention of Cell Death and Loss of RGCs after Ranibizumab Treatment

It is well investigated that ischemia/reperfusion leads to a significant RGC death [11,39,40,41]. We could also observe this effect fourteen days after ischemia on protein level via immunohistology, as well as on the mRNA level. For identification and quantification of the RGCs, we used the transcription factor Brn-3a/*Pou4f1* of the Brn-3 family. It should be noted that, in this case, half of the ipsilateral-RGCs and all of the ip-RGCs were not identified. Nevertheless, Brn-3a is the most common Brn-3 member, its expression level reduces after axonal injury and, therefore, Brn-3a is ideally suited as marker to evaluate RGCs after damage [20]. Interestingly, in our study there was a trend to a higher number of RGCs after treatment with ranibizumab, when applied one day after ischemia/reperfusion, compared to the ischemic group, while significantly fewer Brn-3a^+^ RGCs were noted after bevacizumab treatment. No effect was seen on the mRNA level. The immunohistological data indicate that the VEGF inhibitor ranibizumab seems to have a certain protective effect on this neuronal cell type. Additionally, Joachim et al. could show at a later point in time that significantly more RGCs were present after ranibizumab treatment in relation to ischemic retinae and, in this case, RGC damage can be impaired by ranibizumab therapy [38]. Possibly, in further studies, a higher sample size is needed to verify these findings. Future studies should also investigate an application of the anti-VEGF drug to an earlier point in time, directly after ischemia induction. Perhaps this would optimize the effect of the VEGF inhibitor.

The focus of our study was particularly laid on cell death mechanisms after ischemia, including apoptosis and autophagy. Apoptosis is characterized as a programmed cell death that is also stimulated, for example, by immune cells. Autophagy is an evolutionarily-conserved, intracellular process in which components like misfolded, long-lived proteins are degraded and damaged cell organelles are recycled [42]. Both processes, autophagy, and apoptosis, can be sequentially activated by signal transduction pathways, which were elicited by cell-intrinsic stress [43]. Previous studies could show that ischemia/reperfusion triggers both cell death mechanisms [23,44]. Abcouwer et al. investigated the effect of bevacizumab on apoptosis following retinal ischemic injury with intravitreal injection of the drug before ischemia induction. They could not show any effect of bevacizumab on caspase activation [23]. Additionally, in our studies the anti-VEGF treatment had no impact on the *caspase* expression on mRNA level. Nevertheless, we detected a decline in the number of apoptotic, as well as autophagocytotic cells, including the colocalized LC3BII^+^ and LAMP1^+^ ones, after treatment with both VEGF inhibitors, bevacizumab and ranibizumab, whereby the positive effect was more prominent in the ranibizumab group. This could also be demonstrated by qRT-PCR for *NF-kB* and *LAMP1* mRNA expression concerning apoptosis and autophagocytosis, respectively. Regarding early autophagocytosis, we could not show any effect. Possibly, an earlier point in time after ischemia/reperfusion needs to be investigated, since other studies detected LC3 immunoreactivity already 24 h after ischemia/reperfusion [44,45].

### 3.3. Positive Impact of the Anti-VEGF Therapy on the Microglia and Cone Bipolar Cells

Microglia are assigned to the population of immune cells. Ischemia, inflammatory events, or cellular stress induce the activation and migration of microglia to the location of injury. This was indicated for ischemia, as well as for neuronal injuries, retinopathies, and photoreceptor degeneration [46,47]. In our study, ischemia also led to significantly increased microglia numbers as well as microglia activation in all ischemic groups. However, even here, it appears that there has been a protective or preventive effect of ranibizumab. The Iba1^+^ cell population was less occurrent in the ranibizumab-treated group than in the other ischemic groups. Regarding the activation state of the microglia, even a significant decrease of ED1^+^, as well as Iba1^+^, cells could be observed in ranibizumab-treated eyes in comparison to bevacizumab-treated ones. Huang et al. also noted a marked inhibition of microglia infiltration after injection of VEGF receptor inhibitors against VEGF receptor 1 and 2 [48]. They used a laser-induced choroidal neovascularization mouse model to evaluate the impact of VEGF receptor blockade on microglial infiltration. The effect of receptor inhibition resembles the inhibition of VEGF itself. Since VEGF binding is impeded, the subsequent pathway is disturbed.

In relation to the immunohistological data, we noted a significant up-regulation of *Iba1* and *CD68* mRNA expression levels in all ischemic groups, the untreated, as well as the VEGF-treated, ones. A possible explanation could be that, in a qRT-PCR analysis, the whole retina, as well as the appendices of the microglia, were measured, whereas via immunohistology only the positive cell bodies in the inner layers were counted. 

We made a similar observation regarding the cone bipolar cells. Additionally, here, a protective effect of VEGF inhibition could be observed. After ischemic injury a significant cone bipolar cell loss could be detected, which no longer existed after treatment with anti-VEGF drugs. Zhao et al. noted a decreased number of recoverin^+^ cone bipolar cells only 6 or 12 h after global ischemia [49]. The impact of anti-VEGF treatment on specific inner retinal cells, like the bipolar cells, after an ischemic insult have hardly been studied so far. One study showed that a high-dose of intravitreal VEGF inhibitors (pegaptanib and ranibizumab) affects PKC expression in rod bipolar cells. However, the VEGF drugs in this study were administered in rabbits without a prior retinal injury [37].

## 4. Materials and Methods

### 4.1. Animals

In this study male Brown-Norway rats (7–8 weeks old; Charles River Laboratories, Sulzfeld, Germany) were used. The animal care committee of North Rhine-Westphalia (Germany) approved all experiments of the study, which were performed in accordance with the ARVO statement for the use of animals in ophthalmic and vision research. Rats were housed in a 12-h light-dark cycle under environmentally-controlled conditions with free access to chow and water.

### 4.2. Induction of Ischemia/Reperfusion

To induce retinal ischemia/reperfusion we used an in our group established ischemia/reperfusion model [11,50]. Ischemia was induced for 60 min by elevating the IOP to 140 mmHg. One eye per animal underwent ischemia, while the contralateral eye was used as control.

### 4.3. Treatment with Bevacizumab and Ranibizumab

One day after ischemia induction, the VEGF inhibitors bevacizumab (Avastin 25 mg/mL; Genentech, South San Francisco, CA, USA) and ranibizumab (Lucentis 10 mg/mL; Novartis, Nürnberg, Germany) were injected intravitreally once. The administered dose was 0.05 mg in an applied volume of 5 µL. Therefore, the animals were anesthetized with a cocktail of ketamine/xylazine (100/4 mg/kg). To dilate the ischemic eye, 5% tropicamide was applied. Additionally, the eye was topically anesthetized with conjuncain (Bausch and Lomb, Berlin, Germany). A 32-gauge Hamilton syringe (Hamilton, Reno, NV, USA) and a stereomicroscope (Carl Zeiss Microscopy, Oberkochen, Germany) were used for the intravitreal injection of bevacizumab and ranibizumab. This resulted in four study groups: control (no ischemia induction, no treatment), ischemia (ischemia induction, no treatment), bevacizumab (beva; ischemia induction plus bevacizumab treatment), and ranibizumab (rani; ischemia induction plus ranibizumab treatment).

### 4.4. Electroretinogram Measurements

Fourteen days after ischemia/reperfusion retinal function was monitored using full-field flash electroretinography (HMsERG system; OcuScience LLC, Rolla, MO, USA). Rats were dark adapted before running the electroretinogram (ERG) measurements under dim red light, as previously described in detail [11]. Briefly, scotopic flash ERGs were recorded at 0.1, 0.3, 1, 3, 10, and 25 cd/m^2^. Signals obtained from the corneal surface were amplified, digitized, averaged, and stored using ERGView software (4.380R; OcuScience LLC) for later analysis. Before evaluating the a- and b-wave amplitudes, a 150 Hz filtering of the data was applied. Data were then transferred to a spreadsheet program (Excel; Microsoft Corp., Redmond, WA, USA) following statistical analysis (Statistica V12; Statsoft, Tulsa, OK, USA).

### 4.5. Tissue Collection and Preparation

Fourteen days after ischemia/reperfusion the eyes were removed and processed for immunohistology (*n* = 5–6/group) and qRT-PCR (*n* = 4/group). For immunohistology, the eyes were fixed in 4% paraformaldehyde, incubated in 30% sucrose, embedded in optical cutting temperature medium (Tissue-Tek; Thermo Fisher Scientific, Cheshire, UK), and stored at −80 °C. Retinal cross-sections, 10 µm thick, were prepared with a cryostat (Thermo Fisher Scientific, Walldorf, Germany) for further staining. For qRT-PCR analyses, the retinae were dissected, snap frozen in a lysis buffer with ß-mercaptoethanol (Sigma-Aldrich, Steinheim, Germany) in liquid nitrogen, and stored at −80 °C until RNA extraction.

### 4.6. Immunohistology of Retinal Sections

Immunohistological stainings were performed as previously described [11,38,50,51,52]. To prepare the retinal cross-sections (*n* = 5–6/group) for immunohistochemistry, they were first dried and rehydrated in PBS, followed by blocking in 10–20% appropriate serum with 1% BSA in 0.1% or 0.2% Triton X-100 in PBS. For each staining six retinal sections per eye were incubated with the appropriate primary and secondary antibodies (Table 1) to label RGCs, apoptotic, and autophagocytotic cells as well as (activated) microglia, amacrine, and cone bipolar cells.

DAPI (4′,6-Diamidin-2-phenylindol; Serva Electrophoresis, Heidelberg, Germany) was always added as a nuclear stain. Negative controls were performed for each staining by applying only the secondary antibody. Four pictures per retinal cross-section, two from the periphery and two from the central part at a distance of approximately 300 and 3100 µm dorsal and ventral to the optic nerve, were taken with a fluorescence microscope (Axio Imager M1; Carl Zeiss Microscopy) [11]. All digitalized images were transferred to Corel Paint Shop Photo Pro (V 13; Corel Corp., Fremont, CA, USA), masked, and excerpts were cut out.

Evaluation was executed under masked conditions with ImageJ software (V 1.44p; NIH, Bethesda, MD, USA). The Brn-3a^+^, Bax^+^, LC3BII^+^, LAMP1^+^, Iba1^+^, ED1^+^, parvalbumin^+^, ChAT^+^, and recoverin^+^ cells were counted and averaged for each slice, as previously described [11,38]. Regarding Bax and ED1, the co-localization with Brn-3a or Iba1 was assessed, respectively.

### 4.7. Quantitative Real-Time PCR Analysis of Retinal Tissue

For qRT-PCR analyses the total RNA from the retinal tissue (*n* = 4/group) was extracted and purified using the DyNAmo ColorFlash SYBR Green qPCR Kit (Thermo Fisher Scientific, Waltham, MA, USA) according to the manufacturer’s instructions. Concentration and purity of the extracted RNA was measured via spectrophotometry (BioSpectrometer; Eppendorf, Hamburg, Germany). cDNA was reverse-transcribed with a cDNA-synthesis kit and random hexamer primers (Sigma-Aldrich, Darmstadt, Germany) by utilizing 1 µg of total RNA. qRT-PCR experiments were realized in a PikoReal 96 real-time PCR system (Thermo Fisher Scientific) using SYBR Green. A dilution series of 5–125 ng cDNA served as a basis to calculate the primer efficiencies of each primer set [53]. Ct values of the house-keeping genes *actin* and *cyclophilin* were considered for normalization and relative quantification (Table 2).

### 4.8. Statistics

Histological data are presented as mean ± standard error mean (SEM) and qRT-PCR data as median ± quartile + minimum + maximum. Regarding histology, groups were compared using ANOVA followed by Tukey post-hoc tests (Statistica V13; Dell, Tulsa, OK, USA). For statistical evaluation of relative expression variations in qRT-PCR analyses, data were analyzed by REST^©^ software (QIAGEN GmbH, Hilden, Germany) using a pairwise fixed reallocation and randomization test. *p*-Values below 0.05 were considered statistically significant.

## 5. Conclusions

The comparison of the impact of the two VEGF inhibitors, bevacizumab and ranibizumab, after induction of ischemia/reperfusion was the focus of our study. Ranibizumab showed a more protective effect than the off-label used bevacizumab. A certain protection of the functionality and the availability of retinal ganglion cells could be observed. In addition, a reduction of the late autophagocytosis, apoptosis and microglia activation could be evaluated after treatment. We assume that the different efficacy is due to the smaller molecule size of ranibizumab, which allows a better retinal penetration. Thus, ranibizumab could be a possible option to treat retinal ischemic damage. Nevertheless, further studies are needed to clarify the underlying molecular mechanism.

## Figures and Tables

**Table 1:** List of used primary and secondary antibodies, including cell type, dilution, and company.

Cell Type/Mechanism	Primary Antibody with Dilution	Company	Secondary Anibody with Dilution	Company
RGCs	Anti-Brn-3a, 1:100	Santa Cruz Biotechnology, Heidelberg, Germany	Alexa 488, 1:500	Dianova, Hamburg, Germany
Apoptotic cells	Anti-Bax, 1:100	Abcam, Cambridge, UK	Alexa 555, 1:400	Invitrogen, Darmstadt, Germany
Early autophagy	Anti-LC3BII, 1:100	Cell Signaling, Danvers, MA, USA	Alexa 488, 1:500	Invitrogen
Late autophagy	Anti-LAMP1, 1:100	Abcam	Alexa 555, 1:400	Invitrogen
Microglia	Anti-Iba1, 1:400	Wako Chemicals, Neuss, Germany	Alexa 488, 1:500	Invitrogen
Activated microglia and macrophages	Anti-ED1, 1:200	Millipore, Darmstadt, Germany	Alexa 555, 1:500	Invitrogen
Amacrine cells	Anti-parvalbumin, 1:100	Swant, Marly, Switzerland	Alexa 488, 1:500	Invitrogen
Cholinergic amacrine cells	Anti-ChAT, 1:250	Millipore	Alexa 488, 1:500	Jackson Immuno Research, Cambridgeshire, UK
Cone bipolar cells	Anti-recoverin, 1:1000	Millipore	Alexa 555, 1:400	Invitrogen

**Table 2:** List of primer pairs used for analyses of RGCs, cell death mechanisms, and microglia mRNA expression in control, ischemic, untreated, as well as treated retinae by qRT-PCR. For relative quantification of mRNA levels, the house-keeping genes *actin* and *cyclophilin* were used. The primer sequence (5′–3′), the predicted amplicon size and the primer efficiency are indicated. Abbreviations: b*p* = base pairs, F = forward, R = reverse.

Gene	Primer Sequence (5′–3′)	Amplicon Size	Primer Efficiency
*β-Actin-F*	cccgcgagtacaaccttct	72 bp	1.000
*β-Actin-R*	cgtcatccatggcgaact		
*Cyclophilin-F*	tgctggaccaaacacaaatg	88 bp	1.000
*Cyclophilin-R*	cttcccaaagaccacatgct		
*Bad-F*	caggcagccaataacagtca	68 bp	1.000
*Bad-R*	tacgaactgtggcgactcc		
*Beclin-1-F*	aggatggtgtctctcgaagatt	77 bp	0.969
*Beclin-1-R*	gatcagagtgaagctattagcactttc		
*Brn-3a (Pou4f1)-F*	ctggccaacctcaagatcc	72 bp	1.000
*Brn-3a (Pou4f1)-R*	cgtgagcgactcgaacct		
*Caspase-3-F*	ccgacttcctgtatgcttactcta	70 bp	1.000
*Caspase-3-R*	catgacccgtcccttgaa		
*Caspase-9-F*	cgtggtggtcatcctctctc	81 bp	1.000
*Caspase-9-R*	gagcatccatctgtgccata		
*CD68-F*	ctcacaaaaaggctgccact	60 bp	1.000
*CD68-R*	ttccggtggttgtaggtgtc		
*ChAT-F*	gcctcatctctggtgtgctt	62 bp	1.000
*ChAT-R*	gtcagtgggaagggagtgg		
*Iba1-F*	ctccgaggagacgttcagtt	96 bp	0.855
*Iba1-R*	tttttctcctcatacatcagaatcatcagaat		
*LAMP1-F*	ctgaaggtggggaacaagag	66 bp	1.000
*LAMP1-R*	caggctagaagtggcattca		
*LC3B-F*	tgaatatgagcgaactcatcaag	92 bp	1.000
*LC3B-R*	catgctgtgcccattcac		
*NFκB-F*	ctggcagctcttctcaaagc	70 bp	1.000
*NFκB-R*	ccaggtcatagagaggctcaa		
*P62-F*	ggggaccctgataacatcaa	73 bp	0.976
*P62-R*	ccttgccggctaagatgag		

## Supplementary Materials

The following are available online at http://www.mdpi.com/1422-0067/19/6/1636/s1.
