# Supplementary material for: Fewer Functional Deficits and Reduced Cell Death after Ranibizumab Treatment in a Retinal Ischemia Model
Source: Int J Mol Sci. 2018 May 31;19(6):1636. doi: 10.3390/ijms19061636 (PMC6032266; doi:10.3390/ijms19061636)
Supplement: Supplementary file 1 [file ijms-19-01636-s001.zip › Supplement+ReviewerComments/Revision_Reviewer1_RebuttalLetter_RanibizumabTreatmentRetinalIschemia_MPalmhofSJoachim.docx]

We thank the reviewers for their very helpful comments and appreciate their gracious review of our manuscript. We have revised our manuscript as per the reviewers’ suggestions and were able to address all issues raised. All manuscript changes are highlighted in red. We hope that the revision will make this manuscript suitable for publication in the *International Journal of Molecular Science*.

**Reviewer 1**

Comments and Suggestions for Authors

The purpose of this study was to compare the protective effects of intravitreal injection of 2 VEGF inhibitors, bevacizumab (“beva”, Avastin, Genetech) and ranibizumab (“rani”, Lucentis. Norvartis) after retinal ischemia. These VEGF inhibitors are in wide used for treatment against “wet” AMD,.

Analysis was done 2 weeks after injection of a single dose of either “beva” or “rani”. Both drugs were injected at the same dosage of 0.05 mg/5 uL although “rani” is about ten times more effective as “beva”.  The VEGF inhibitors were injected one day after ischemia/reperfusion injury. Two week after ischemia/reperfusion, retinal function was tested by electroretinography (Scotopic flash ERGs were recorded at 0.1, 0.3, 1, 3, 10, and 25 cd/m2). Retinae were analyzed by immunohistochemistry of ganglion cell and other retinal cell markers, markers for early and late cell death, and qPCR for the mRNA expression of Pouf4 (ganglion cells), Bad (apoptosis), LAMP1 (late autophagy), Iba1 (microglia), CD68 (activated microglia), and ChAT (amacrine cells).

The study shows that “rani” was partially protective against ischemia (at 2 weeks post ischemia) whereas “beva” mostly had no effect.

The same group has previously published a study about the effects of “rani” 3 weeks after ischemia/reperfusion.

General comments:

This is overall a well-designed study, looking at different many aspects of the model.

1)     One concern is why the authors decided to use the same dosage for both “rani” and “beva” although there is such a difference in efficacy. Maybe they should explain why these dosages were chosen.

Response: We thank the reviewer for pointing this out. We agree with the reviewer, that the efficacy of the two VEGF inhibitors is different. However, we wanted to draw a comparison when using the same dose. Since we have seen positive and protective effects of ranibizumab in previous studies of our group by applying 0.05 mg (Joachim et al., 2017), we wanted to keep this dose also for the comparative study. This allows to make a possible comparison regarding the beginning of therapy. In addition, we consulted previous publications that also used this dose and could determine corresponding effects (Abcouwer et al., 2010; *Effects of ischemic preconditioning and bevacizumab on apoptosis and vascular permeability following retinal ischemia-reperfusion injury.* Invest Ophthalmol Vis Sci; 51(11): 5920-33). Lu and Adelman calculated for administration of the VEGF inhibitors in rats an equivalent dose to human dose, since the volume of rat vitreous varies from 12 µl to 56 µl and the human vitreous volume comprises about 4 ml (Lu and Adelman, 2009; *Are intravitreal bevacizumab and ranibizumab effective in a rat model of choroidal neovascularization.* Graefes Arch Clin Exp Ophthalmol; 247: 171-177).

2)     The reviewer has one question about the analysis of immuno-stained tissue sections: Four pictures were taken from each retinal cross section (2 peripheral and 2 central). Did the sections include the optic nerve, or were they just outside? Were the sections oriented superior-inferior, or nasal-temporal?

Response: We apologize for the missing of a precise description regarding the image acquisition. For each immunohistological staining, we analyzed four pictures per retinal cross-section. The pictures were taken at a distance of approximately 300 and 3100 µm dorsal and ventral to the optic nerve, as described in a previous study of the ischemia-reperfusion model (Schmid, et al., 2014; *Loss of inner retinal neurons after retinal ischemia in rats.* Invest Ophthalmol Vis Sci; 55, 2777-2787). The optic nerve itself was not included. Pictures were acquired with a fluorescence microscope (Axio Imager M1 and M2; Carl Zeiss Microscopy) using a 400x magnification. Each original picture had a size of 1388x1040 pixel, which corresponds to a size of 217.12x162.69 µm. Of each picture we prepared equal cut outs of a defined area of the retina (800x600 pixel; 125.14x93.86 µm).

Regarding the orientation of the sections, we investigated the peripheral and central retina both nasal-temporal and superior-inferior. Since we analyzed six cross-sections per eye, we chose three from the periphery and three from the center of the eye, which corresponds to superior-inferior. Ischemia was induced homogeneously, which allowed us to consider each area of the retina as rather equivalent.

3)     One correction: Recoverin labels cone bipolar cells and photoreceptors (not rod bipolar cells). The marker for rod bipolar cells would be PKCα.

Response: We thank the reviewer for this indication. This was corrected in the appropriated sections.
