# Supplementary material for: Fewer Functional Deficits and Reduced Cell Death after Ranibizumab Treatment in a Retinal Ischemia Model
Source: Int J Mol Sci. 2018 May 31;19(6):1636. doi: 10.3390/ijms19061636 (PMC6032266; doi:10.3390/ijms19061636)
Supplement: Supplementary file 1 [file ijms-19-01636-s001.zip › Supplement+ReviewerComments/Revision_Reviewer2_RebuttalLetter_RanibizumabTreatmentRetinalIschemia_MPalmhofSJoachim.docx]

We thank the reviewers for their very helpful comments and appreciate their gracious review of our manuscript. We have revised our manuscript as per the reviewers’ suggestions and were able to address all issues raised. All manuscript changes are highlighted in red. We hope that the revision will make this manuscript suitable for publication in the *International Journal of Molecular Science*.

**Reviewer 2**

Comments and Suggestions for Authors

*Palmhof et al* have provided interesting and stimulating data about the effect of two anti-VEGFs: **ranibizumab and bevacizumab** on retinal cell types after ischemia/reperfusion. According to that, the title of the manuscript should be changed or modified since the authors have found some effects of the bevacizumab on b-wave, Brn3a-RGCs density, Pou4f1, LAMP1 expression etc.

Response: We thank the reviewer for this comment. As suggested by the reviewer, we modified the title to “Less functional deficits and reduced cell death after ranibizumab treatment in a retinal ischemia model”. We hope that the new title is now more appropriate.

The manuscript has an appropriate structure and language. The data are compelling but major revision will greatly improve the report.

**Results:**

-Add in the figure legends the abbreviations for Beva: bevacizumab and Rani: ranibizumab.

Response: We apologize for the missing abbreviations. We added the explanation of the treatment substances to all figure legends.

-Line 8 (page 3). RGCs was mentioned previously in the manuscript. Revise.

Response: As suggested by the reviewer, the manuscript was revised regarding the first time use of the abbreviation for retinal ganglion cells (RGCs).

-Line 9 (page3). Add “Forty days” instead “14 days”. Revise all the manuscript.

Response: We thank the reviewer for this advice. We revised the whole manuscript and replaced “14 days” by “fourteen days”.

-Figure 2B and C. Why figure 2B is expressed in cells/mm and figure 2C in percentage? Unify.

Response: We thank the reviewer for pointing this out. For a uniform presentation, we are displaying the Brn-3a occurrence in figure 2B in percentage now. The results part was adjusted accordingly.

-Please, provide a supplementary figure that show the entire retinal section at low magnification (indicate all the retinal layers) in order to check the structure of the RPE, ONL, OPL, INL after ischemia and treatments with all markers used. For example in the case of Figure 5A, is important to observe the immunolabeling of parvalbumin and recoverin in the inner plexiform layer. Is it modified by the ischemia and treatments?

Response: We thank the reviewer for this advice. We prepared a supplementary figure with exemplary pictures of the whole retina for all markers used in the immunohistochemistry (Supplement Figure 1). Retinal cross-sections of all groups, control, ischemia, bevacizumab, and ranibizumab treated ones, were stained with anti-Brn-3a for RGCs, anti-Bax for apoptotic cells (Supplement Figure 1A), anti-LC3BII for early, and anti-LAMP1 for late autophagy (Supplement Figure 1B). RGCs and apoptotic cells were localized in the GCL. Also, positive autophagy signals were mainly present in the GCL. Furthermore, microglia were marked using anti-Iba1 and activated microglia with anti-ED1 (Supplement Figure 1C). Microglia as well as their activated type were observed in the GCL, IPL, and INL. AII amacrine cells were labeled with anti-parvalbumin and cholinergic amacrine cells with anti-ChAT, while cone bipolar cells were visualized with anti-recoverin (Supplement Figure 1D). Marked cell bodies of AII and cholinergic amacrine cells as well as cone bipolar cells were noted in the INL. Furthermore, some displaced cholinergic amacrine cells were visible in the GCL. Regarding ChAT immunoreactivity, a distinct stratification in the IPL was observed in the control group and a weakened one in the ranibizumab treated group, while it was not present after ischemia induction and bevacizumab treatment. Also, immunolabeling of parvalbumin was noted in the IPL of control retinae, but not in the ischemic and anti-VEGF treated groups. No recoverin immunolabeling was detected in the IPL of all groups. We also added this observations to the corresponding results.

-In order to analyze the amacrine cells after ischemia and treatments, the authors used parvalbumin by immunostaining and *ChAT* by qRT-PCR, why the authors did not immunofluorescence with ChAT antibodies?

Response: We thank the reviewer for pointing this out. As suggest by the reviewer, we performed an additional staining with an anti-ChAT antibody. The evaluation was added to figure 5 (Figure 5A: exemplary pictures of the ChAT immunostaining; Figure 5D: statistical analysis of the cell count). The evaluation of the ChAT immunostaining confirmed the results of the parvalbumin staining. Also here, counting of ChAT^+^ cells revealed a significant loss of the amacrine cells in the ischemic (p<0.001), the bevacizumab (p<0.001), and ranibizumab treated group (p<0.001) in comparison to control retinae. These findings were added to the results section.

**Discussion**

The authors have to take into account in the discussion some aspects:

1) Brn3 members (Brn-3a, Brn-3b and Brn-3c) are expressed in RGCs and when using Brn3a marker to identify RGCs, the RGC sub-populations that are not being detected are half of the ipsilateral-RGCs and all of the ip-RGCs. Furthermore the level of Brn3a expression in the surviving RGCs decrease after axonal injury. Please, according to these data, add the following reference: *Nadal-Nicolás et al., 2012. Whole number, distribution and co-expression of brn3 transcription factors in retinal ganglion cells of adult albino and pigmented rats. PLoS One. 2012;7(11):e49830*.

Response: We thank the reviewer for this advice and added the corresponding reference in the results part where we mentioned Brn-3a as specific RGC marker. Furthermore, we mentioned this fact in the discussion: “For identification and quantification of the RGCs, we used the transcription factor Brn-3a/*Pou4f1* of the Brn-3 family. It should be noted, that in this case half of the ipsilateral-RGCs and all of the ip-RGCs were not identified. Nevertheless, Brn-3a is the most common Brn-3 member, its expression level reduces after axonal injury and therefore Brn-3a is ideally suited as marker to evaluate RGCs after a damage.”

2) Recoverin marker label rat cone bipolar cells, the authors should indicate it.

Response: We thank the reviewer for this indication. We have adapted this throughout the manuscript.

3) It is necessary to explain in the discussion why the authors have decide to analyze Bad/Bax molecules in order to investigate the apoptotic effect (why not caspase-3 or TUNEL). Bax is a pro-apoptotic mitochondrial marker pathway and it is has been reported that the death or survival is determined by levels of pro-apoptotic in each cell. Clarify it.

Response: We thank the reviewer for this advice. We agree with the reviewer, that Bad and Bax are proteins of the pro-apoptotic mitochondrial pathway. As suggested by the reviewer and in order to investigate also the extrinsic pathway, we additionally analyzed *caspase-3*, *caspase-9*, and *NFkB* expression on mRNA level. The corresponding data were added to figure 2F-H. Evaluation of the relative *caspase-3* mRNA expression displayed a significant up-regulation in ischemic (p=0.026), bevacizumab (p<0.001), and ranibizumab treated retinae (p=0.009), when compared to control eyes. Regarding expression of relative *caspase-9* mRNA, no differences in the expression level could be measured between the control and all ischemic eyes (ischemia: p=0.131, beva: p=0.118, rani: p=0.858). However, analysis of relative *NFkB* mRNA expression revealed a significant elevation in the ischemic (p=0.034) and bevacizumab treated group (p=0.008) in comparison to control retinae. No differences were seen between the control and ranibizumab treated retinae (p=0.069). These findings were added to the results and discussion section.

4) The authors describe the results and observe certain preservation of the neuronal function, however, a hypothesis of the neuroprotection (preservation in the function and density of the inner neurons in the INL) is demanded. (p.e are there VEGF receptor in RGCs/amacrine cells?)

Response: Several previous studies could demonstrate, that inflammatory events and ischemia induce a VEGF response in the retina (Cervia et al., 2012; *Vascular endothelial growth factor in the ischemic retina and its regulation by somatostatin.* Journal of neurochemistry; 120(5): 818-29. Bates et al., 2003; *The role of vascular endothelial growth factor in wound healing*. Int J Low Extrem Wounds; 2(2): 107-20. Abcouwer et al., 2010; *Effects of ischemic preconditioning and bevacizumab on apoptosis and vascular permeability following retinal ischemia-reperfusion injury.* Invest Ophthalmol Vis Sci; 51(11): 5920-33). In human retina, VEGF is expressed by all major classes of neurons. Especially, in cell bodies of amacrine cells and RGCs immunoreactivity of VEGF is found *(*Famiglietti et al., 2003; *Immunocytochemical localization of vascular endothelial growth factor in neurons and glial cells of human retina.* Brain Res; 969(1-2): 195-204). Also, in the rat retina immunoreactivity of VEGF can be verified in the GCL and INL. Here, a predominant localization of VEGF is assumed in neurons, while in retinal vessels it is scarcely localized (Lee et al., 2007; *Short-term hyperhomocysteinemia-induced oxidative stress activates retinal glial cells and increases vascular endothelial growth factor expression in rat retina.* Biosci Biotechnol Biochem; 71(5): 1203-10). Therefore, it was of great interest to investigate the possible effect of VEGF inhibition on RGCs and amacrine cells. We also added this information to the discussion.

5) In previous studies of your group: *Joachim et al., 2017. Protective effects on the retina after ranibizumab treatment in an ischemia model. PLoS One. 2017 Aug 11;12(8):e0182407*, the authors demonstrated that photoreceptors and RGCs are protected by ranibizumab treatment but amacrine cells cannot be rescued. Is it a contradiction with the results of this manuscript?

Response: We thank the reviewer for this comment. We agree with the reviewer, that in the mentioned previous manuscript our group could show a significant protection of photoreceptors and RGCs by ranibizumab treatment. But this is not a contradiction with the current results. In the previous studies, treatment with ranibizumab was performed three days after ischemia induction, in this manuscript the VEGF inhibitors were injected one day after ischemia induction. In addition to the comparison of the different inhibitors, we also wanted to compare the time aspect of the therapy. Also, the current data demonstrated, that amacrine cells cannot be rescued indicating how sensitive this cell type is to an ischemia induction. Regarding photoreceptors, we performed ERG measurements to investigate the functionality of the neurons. The a-wave amplitude displays the activity of the photoreceptors. Here, we could measure a tendentially higher amplitude in ranibizumab treated eyes comparing to ischemic ones, which would fit with the previous data. Concerning RGCs, the RGC loss in ranibizumab treated retinae was not as prominent as in untreated ischemic or bevacizumab treated eyes. Thus, we were also able to show a certain protective effect on this cell type after ranibizumab therapy by applying treatment earlier.

6) The authors analyze retinal section from periphery and central retina, however, the results have not been divided by areas. Note that the distribution and density of the rat RGCs is different (*Reese BE, Cowey A. Large retinal ganglion cells in the rat: their distribution and laterality of projection. Exp Brain Res. 1986;61(2):375-85)  Urcola et al., 2006. Three experimental glaucoma models in rats: comparison of the effects of intraocular pressure elevation on retinal ganglion cell size and death. Exp Eye Res. 2006 Aug;83(2):429-37) (Danias et al., 2002. Cytoarchitecture of the retinal ganglion cells in the rat. Invest Ophthalmol Vis Sci. 2002 Mar;43(3):587-94*).

Response: We thank the reviewer for this advice. We agree with the reviewer, that the distribution and density of RGCs is different. The number of RGCs in the rat retina decreases from the center to the periphery. For the evaluation, we counted the RGCs in the peripheral as well as central areas of the cross-sections and subsequently averaged the cell number for each slice as previously described (Schmid, et al., 2014; *Loss of inner retinal neurons after retinal ischemia in rats.* Invest Ophthalmol Vis Sci; 55, 2777-2787; Joachim et al., 2017; *Protective effects on the retina after ranibizumab treatment in an ischemia model.* PLoS One, 12; Kuehn et al., 2017; *Concentration-dependent inner retina layer damage and optic nerve degeneration in a nmda model.* J Mol Neurosci, 63, 283-299). Thus, the distribution and occurrence of RGCs is reflected throughout the retina.

-Line 20 (page 10). Indicate that the toxicity of the anti-VEGFs has been investigated *in vivo* and *vitro* (For example: *Saenz-de-Viteri et al., 2016. Single- and repeated-dose toxicity study of bevacizumab, ranibizumab, and aflibercept in ARPE-19 cells under normal and oxidative stress conditions Biochem Pharmacol. 1;103:129-39*).

Response: As suggested by the reviewer, we have included this indication in the context. We have added the information that the toxicity of VEGF inhibitors has been evaluated *in vivo* and *in vitro* and also added the mentioned reference to the discussion section.

**Materials and Methods**

-Please,it is advisable to use **male and female** animals in preclinical studies in your future experiments (*Clayton JA, Collins FS. Policy: NIH to balance sex in cell and animal studies. Nature. 2014 May 15;509(7500):282-3*).

Response: We thank the reviewer for this advice. We will implement female and male animals in future studies.

-Please, specify the distance, area (temporal, ventral) of the picture captured in the retina in the immunofluorescence sections.

Response: We thank the reviewer for this indication. The pictures we analyzed for each immunohistological staining were taken at a distance of approximately 300 and 3100 µm dorsal and ventral to the optic nerve, as described in a previous study of the ischemia-reperfusion model (Schmid, et al., 2014; *Loss of inner retinal neurons after retinal ischemia in rats.* Invest Ophthalmol Vis Sci; 55, 2777-2787). We added this explanation to the Materials and Methods section.

-The majory of the articles used anti-VEGFs at clinical doses. Why have the authors chosen these doses?

Response: We thank the reviewer for pointing this out. We agree with the reviewer, that the majority of articles use the VEGF inhibitors at clinical doses. Since we have seen positive and protective effects of ranibizumab in previous studies of our group (Joachim et al., 2017) by applying 0.05 mg, we wanted to keep the same dose also for the comparative study. This allows us to make a possible comparison regarding the beginning of therapy. Concerning the different VEGF inhibitors, we wanted to draw a comparison at the same dose. In addition, we consulted previous publications that also used this dose by calculating an equivalent dose to human dose for administration of the VEGF inhibitors in rats. It should be noted that the volume of rat vitreous varies from 12 µl to 56 µl, while the volume of human vitreous comprises about 4 ml (Lu and Adelman, 2009; *Are intravitreal bevacizumab and ranibizumab effective in a rat model of choroidal neovascularization.* Graefes Arch Clin Exp Ophthalmol; 247: 171-177).

-Line 29 (page 12), provide the reference of "conjuncain".

Response: We thank the reviewer for this advice and apologize for the missing reference for conjuncain. We have added these.
