# Supplementary material for: Fewer Functional Deficits and Reduced Cell Death after Ranibizumab Treatment in a Retinal Ischemia Model
Source: Int J Mol Sci. 2018 May 31;19(6):1636. doi: 10.3390/ijms19061636 (PMC6032266; doi:10.3390/ijms19061636)
Supplement: Supplementary file 1 [file ijms-19-01636-s001.zip › Supplement+ReviewerComments/Revision_Reviewer3_RebuttalLetter_RanibizumabTreatmentRetinalIschemia_MPalmhofSJoachim.docx]

We thank the reviewers for their very helpful comments and appreciate their gracious review of our manuscript. We have revised our manuscript as per the reviewers’ suggestions and were able to address all issues raised. All manuscript changes are highlighted in red. We hope that the revision will make this manuscript suitable for publication in the *International Journal of Molecular Science*.

**Reviewer 3**

Comments and Suggestions for Authors

This paper by Marina Palmhof and colleagues reports protective effects of the VEGF-blocking agent ranimizumab against functional and anatomic damages induced by ischemia-reperfusion in rat retinas. The effects of ranimizumab are compared with a similar VEGF-binding molecule, bevacizumab. Different types of analysis are performed, including ERG and markers of apoptosis and of autophagy, markers of specific neuronal populations, microglia markers, evaluated both with immunohistochemistry and qPCR. Overall, a quite extensive evaluation of different parameters has been performed and the results indicate a small, but in most cases statistically significant, protective effect of ranimizumab, which appears to be a better protectant than bevacizumab.

Although, the experimental procedures are simple and appropriate and the results are clearly presented, there are at least three major problems that, in my opinion, affect this paper.

First, this paper is very similar to the paper by researchers of the same group listed as ref n. 21. The differences include a different survival time after ischemia/reperfusion (21 days in the previous paper, 14 days in the present paper) and the evaluation of ERG and of autophagy markers in the present paper; however the main point is identical and it consists in the demonstration that ranimizumab protects retinal neurons from ischemia/reperfusion damage. I am not sure that the present paper was needed to confirm this observation.

Response: We agree with the reviewer, that also in the mentioned previous manuscript of our research group the effect of ranibizumab treatment on ischemic retina was investigated. The main difference between the previous study and the current manuscript is on the one hand the beginning of the therapy and comparison of two different VEGF inhibitors and on the other hand the focus of examinations. In the previous studies, treatment with ranibizumab was performed three days after ischemia induction, while in this manuscript the VEGF inhibitors were injected one day after ischemia induction. This allows an assessment concerning the best point in time to start therapy with VEGF inhibitors after ischemic injury. In addition, in the current study we performed a comparison of two different inhibitors with the focus on cell death mechanisms. The impact of these anti-VEGF drugs is still part of clinical discussion. Thus, the present manuscript should contribute to a better understanding of the mode of action of both inhibitors.

Second, I have problems in understanding the rationale of this investigation. At a first impression, it seems that the Authors propose VEGF blockade as a neuroprotective strategy. In fact, both bevacizumab and ranimizumab are monoclonal antibodies designed to inhibit VEGF effects on blood capillaries. They are applied in the treatment of retinopathies to cure or to prevent vascular damage and the proliferative phase, that is to prevent the effects of VEGF on the vascular tissue of the retina. In the presence of vascular damage, the disruption of the blood-retina barrier causes changes in the microenvironment that may lead to neuronal death. Therefore, there is no direct action of VEGF against neurons, as the Authors seem to imply. The blockade of VEGF may be expected to be protective to neurons only after some time from the beginning of the pathologic state, that is when the vascular damages provoked by VEGF start to affect neuronal survival. So, what is the rationale of using a VEGF-blocking agent to protect neurons in early phases of the pathology (no terrible vascular changes are expected to appear within 14 days)? On the other hand, there are several reports indicating VEGF as a neuroprotective factor in the retina, but the Authors do not mention this fact and do not take into consideration possible positive effects of VEGF on neuronal survival. In addition, there is no discussion to explain or to propose a mechanisms by which VEGF blockade would protect neurons from ischemia-induced death. In summary: i) VEGF is not toxic to neurons; ii) VEGF is neuroprotective; iii) vascular damages within 14 days of ischemia/reperfusion may be not extensive – therefore, what is the rationale for expecting protective effects of ranimizumab; and, since some (limited) effects are indeed observed, how could they be explained?

Response: We thank the reviewer for this comment. We agree with the reviewer, that an increased VEGF expression leads to pathological conditions, like angiogenesis, increased vascular permeability, and further inflammatory responses. It is also known, that inflammatory events and ischemia induce a VEGF response in the retina. The impact of VEGF seems to be dose-dependent (Cervia et al., 2012; *Vascular endothelial growth factor in the ischemic retina and its regulation by somatostatin.* Journal of neurochemistry; 120(5): 818-29; Bates et al., 2003; *The role of vascular endothelial growth factor in wound healing*. Int J Low Extrem Wounds; 2(2): 107-20; Abcouwer et al., 2010; *Effects of ischemic preconditioning and bevacizumab on apoptosis and vascular permeability following retinal ischemia-reperfusion injury.* Invest Ophthalmol Vis Sci; 51(11): 5920-33). Besides its expression by vascular endothelial cells, VEGF is also expressed by all major classes of neurons in the human retina. Especially, in cell bodies of amacrine cells and RGCs immunoreactivity of VEGF is found *(*Famiglietti et al., 2003; *Immunocytochemical localization of vascular endothelial growth factor in neurons and glial cells of human retina.* Brain Res; 969(1-2): 195-204). Also, in the rat retina immunoreactivity of VEGF is prominent in the GCL and INL. Here, a predominant localization of VEGF is assumed to be in neurons, while in retinal vessels it is scarcely localized (Lee et al., 2007; *Short-term hyperhomocysteinemia-induced oxidative stress activates retinal glial cells and increases vascular endothelial growth factor expression in rat retina.* Biosci Biotechnol Biochem; 71(5): 1203-10). Furthermore, studies could show, that ischemia affects retinal VEGF and its expression increased in neurons after ischemia induction (Cervia et al., 2012; *Vascular endothelial growth factor in the ischemic retina and its regulation by somatostatin.* Journal of neurochemistry; 120(5): 818-29; Shima et al., 1996; *Cloning and mRNA expression of vascular endothelial growth factor in ischemic retinas of Macaca fascicularis.* Invest Ophthalmol Vis Sci.; 37(7): 1334-40). We also agree with the reviewer, that VEGF has neuroprotective properties, which was already examined by various studies. Nevertheless, its neurodegenerative characteristics are also discussed and the neurodegenerative effect of VEGF on retinal neurons is not well investigated. The role of VEGF in the nervous system is not only limited to the regulation and development of vessel growth, VEGF also acts directly on different retinal neurons and cause its damage (Carmeliet et al., 2013; *VEGF ligands and receptors: implications in neurodevelopment and neurodegeneration.* Cell Mol Life Sci.; 70(10): 1763-78; Iwona, 2016; *Growth Factors in the Pathogenesis of Retinal Neurodegeneration in Diabetes Mellitus.* Curr Neuropharmacol.; 14(8): 792-804). Therefore, it was of great interest to investigate the effect of VEGF inhibition on inner retinal neurons, like RGCs and amacrine cells, including the comparison of the two different inhibitors. The mechanisms underlying neuronal degeneration and possible protection by VEGF inhibition are still unclear and need to be addressed and investigated in future studies. By examining various cell death mechanisms in the current manuscript, we have made a first step into this direction.

If the hypothesis is that VEGF blockade protects neurons because it limits the vascular damage, then data should be provided showing the amelioration of the vascular pathology in the presence of ranimizumab, paralleled by neuronal survival and efficiency.

Response: We agree with the reviewer, that protection of neurons by VEGF blockage due to limitation of the vascular damage is one possible hypothesis. However, we have followed a different approach. Since VEGF is expressed by neuronal cells in the GCL and INL and it could be shown, that its expression in neurons is increased after ischemia, it is to assume that VEGF blockage protects neurons by intervening in the cell death mechanism. Therefore, one aspect of the current study was to evaluate the impact of VEGF inhibition on apoptosis and autophagy to get first perceptions. How this interaction looks like in detail and which molecular mechanism it is based on has to be determined in further studies.

Third, the assessment of autophagy does not seem to be very accurate. Indeed, in general it is assumed that cells undergoing autophagy should demonstrate the co-localization of p62, LC3, and lysosomal markers (such as LAMP1), or at least autophagy should be demonstrated using more than a simple marker. In addition, it is not clear why “early autophagy” markers, like LC3, do not change, while “late autophagy” markers are instead showing some changes. The Authors should give some interpretation of this finding. Finally, both apoptosis and autophagy have been observed to increase following ischemia, but, again, the Authors do not provide any interpretation for this observation. For instance, autophagy may initiate a cell protective response or may function as a cell death route (but then, why the apoptotic route is also activated?). Also, it is known that in other cases autophagy would reduce the propensity of cells to undergo apoptosis, while apoptotic signaling, in turn, would inhibit autophagy. How come that in this case both processes are activated, and how ranimizumab could reduce both?

Response: We thank the reviewer for this advice. To investigate the autophagocytotic process, especially the activation, more in detail, we performed additional qRT-PCR analyses with the markers *LC3B*, *p62*, and *beclin-1* for indication of autophagy. Furthermore, we evaluated the colocalization of LC3BII^+^ and LAMP1^+^ cells immunohistologically. The corresponding findings were added to the results part (Figure 3D, E, H, I). No differences in *LC3B* mRNA expression were detected comparing the control group and the ischemic (p=0.46), bevacizumab (p=0.625), and ranibizumab treated retinae (p=0.38; Figure 3E). Regarding relative *p62* expression, the ischemic (p=0.968), the bevacizumab (p=0.314) and ranibizumab treated retinae (p=0.748) displayed a similar *p62* level in comparison to the control group (Figure 3H). Equally, no differences in *beclin-1* mRNA expression could be detected between the control group and all ischemic retinae, without (p=0.888) and with treatment (beva: p=0.086, rani: p=0.178; Figure 3I). Investigation of colocalized LC3BII^+^ and LAMP1^+^ cells revealed a significant increase in the ischemia (p=0.007) and bevacizumab treated group (p<0.001) in comparison to the control group, but not in eyes which received treatment with ranibizumab (p=0.2; Figure 3D). However, a significant decrease of LC3BII^+^ and LAMP1^+^ cells was detected in ranibizumab treated eyes in comparison to bevacizumab treated ones (p=0.02). The turnover of organelles, proteins within cells, and of cells within organisms is controlled by both mechanism, autophagy and apoptosis. There are many stress pathways, which consecutively cause autophagy and apoptosis within the same cell (Marino et al., 2014; *Self-consumption: The interplay of autophagy and apoptosis.* Nat Rev Mol Cell Biol, 15, 81-94.). We agree with the reviewer, that in general autophagy inhibits apoptosis induction and apoptosis activation the initiation of autophagy in reverse. However, in special cases, autophagy or autophagy-relevant proteins can affect apoptosis and lead to an activation, so that both mechanisms are activated (Marino et al., 2014; *Self-consumption: The interplay of autophagy and apoptosis.* Nat Rev Mol Cell Biol, 15, 81-94). Previous studies could demonstrate, that ischemia induction elicits both mechanisms, apoptosis and autophagy (Abcouwer et al., 2010; *Effects of ischemic preconditioning and bevacizumab on apoptosis and vascular permeability following retinal ischemia-reperfusion injury.* Invest Ophthalmol Vis Sci, 51, 5920-5933; Dvoriantchikova et al., 2014; *Retinal ganglion cell (rgc) programmed necrosis contributes to ischemia-reperfusion-induced retinal damage.* Exp Eye Res, 123, 1-7; Wei et al., 2015; *Activation of autophagy and paraptosis in retinal ganglion cells after retinal ischemia and reperfusion injury in rats.* Exp Ther Med, 9, 476-482; Piras et al., 2011; *Activation of autophagy in a rat model of retinal ischemia following high intraocular pressure.* PLoS One, 6, e22514). We could show, that ischemia/reperfusion triggers both mechanisms simultaneously and VEGF inhibition diminished both processes. How the interaction between VEGF and cell death works in detail must be investigated in further studies. Regarding the early autophagy, we suppose that it needs to be investigated at an earlier time in point after ischemia/reperfusion. Piras et al. and Wei et al. detected LC3 immunoreactivity as early as 24 h after ischemia/reperfusion.
