# Supplementary material for: Fewer Functional Deficits and Reduced Cell Death after Ranibizumab Treatment in a Retinal Ischemia Model
Source: Int J Mol Sci. 2018 May 31;19(6):1636. doi: 10.3390/ijms19061636 (PMC6032266; doi:10.3390/ijms19061636)
Supplement: Supplementary file 1 [file ijms-19-01636-s001.zip › Supplement+ReviewerComments/Revision_Reviewer4_RebuttalLetter_RanibizumabTreatmentRetinalIschemia_MPalmhofSJoachim.docx]

We thank the reviewers for their very helpful comments and appreciate their gracious review of our manuscript. We have revised our manuscript as per the reviewers’ suggestions and were able to address all issues raised. All manuscript changes are highlighted in red. We hope that the revision will make this manuscript suitable for publication in the *International Journal of Molecular Science*.

**Reviewer 4**

Comments and Suggestions for Authors

First of all I want to congratulate the authors on their manuscript. The study is well designed and manuscript is of interest.

However, several points need to be addressed:

First, language editing is necessary throughout the text. Words are used wrong several times, tense is used in a confusing way and especially “the” is missing or used at the wrong spot. Especially the Introduction needs to be carefully revised. Here, some formulations need to be more precise E.g. Page 2 line 8 ….ranibizumab specifically binds….

Response: We thank the reviewer for this helpful comment. We apologize for any grammar or language errors. All mentioned errors were corrected and the appropriate paragraphs rewritten. We also carefully proofread the whole manuscript and hope these improvements facilitate reading our manuscript.

Page 2 line 15 Calling Aflibercept the third drug is wrong. Also “VEGF trap molecule” is incorrect

Response: We thank the reviewer for this advice and apologize for the ambiguous term. We have rewritten the corresponding sentence.

Page 2 Line 22: “…of both…” is misleading

Response: We thank the reviewer for this advice. We have rewritten this part.

Page 3 Line 12: Needs to be: Quantification of the immuno….

Response: As suggest by the reviewer, we have corrected this.

That ERGs were performed is missing, when the methods are mentioned.

Response: We thank the reviewer for this advice and apologize for the missing listing of the ERG measurements. We have revised this and added, that ERGs were performed.

Most important, it should be briefly described either at the End of the Introduction or at the beginning at the Results how the experiments were performed: day of injection, dose, study time. This is all mentioned in M&M but this is the last chapter. It would be nice if the main parameters are also mentioned before.

Response: As suggested by the reviewer, we briefly described the main parameters of the study (dose, day of injection, study time) at the end of the introduction.

The Results part needs 1-2 sentences more at the beginning.

Response: We added a few sentences at the beginning of the results part. “The comparison of the two VEGF inhibitors bevacizumab and ranibizumab was the main aspect of our study. The findings should contribute to a better understanding of the mode of action of the two different inhibitors.” We hope, that this contributes to a better understanding.

For ease of reading is should also be mentioned that Ranbizumab and Bveacizumab treated eyes also received Ischemia. It is logic, but never mentioned in the Results part. Only with one word in the Introduction.

Response: As suggested by the reviewer, we added a few sentences at the beginning of the results section and mentioned that anti-VEGF treated eyes also underwent ischemia. “Thus, we had three ischemic groups for the investigations, one ischemia only and two groups, which additionally received anti-VEGF treatment. These groups were compared to a control group.” We hope, that this contributes to an easier reading of the manuscript.

Figure 2D: The authors should write Brn3a expression on the y-Axis as they always use that in the text.

Response: As suggested by the reviewer, the y-Axis in figure 2D has been adjusted accordingly.

*Bad* should be explained explained, what it is.

Response: We thank the reviewer for this advice. We added an explanation to the results part, where *Bad* is mentioned the first time.

I think it is a bit ambitious to state that especially apoptosis was studied, if only two apoptotic markers were checked. Additional markers would improve the statement.

Response: We agree with the reviewer, that more markers are needed to make an accurate statement about the apoptosis process. Therefore, we performed additional qRT-PCR analyses with markers for the extrinsic apoptotic pathway. We measured the relative mRNA expression of *caspase-3*, *caspase-9*, and *NFkB*. The corresponding data are shown in figure 2F-H. Analysis of the relative expression of *caspase-3* mRNA displayed a significant elevation of *caspase-3* mRNA levels in the ischemic (p=0.026) and the bevacizumab (p<0.001) and ranibizumab treated group (p=0.009), when compared to control retinae. No differences in expression levels could be detected between the control and all ischemic retinae (ischemia: p=0.131, beva: p=0.118, rani: p=0.858) regarding expression of relative *caspase-9* mRNA. However, evaluation of relative *NFkB* mRNA expression revealed a significant up-regulation in the ischemic (p=0.034) and bevacizumab treated group (p=0.008) in comparison to control retinae. No differences were measured between control and ranibizumab treated retinae (p=0.069). These findings were also added to the results and discussion part.

The most interesting effect would have been if one of the VEGF inhibitors would have caused a significant difference to the ischemia only eyes. However, this only occurred for the b-wave of the ERG with Ranibizumab. Other than that, there was only no significant difference to the control. The fact that there was no difference to the ischemia treated eyes should be mentioned (more often).

Response: We thank the reviewer for this comment. We revised the manuscript and mentioned that there were no differences comparing the anti-VEGF treated groups to the ischemia treated eyes.

N=? should be given in figure (legends).

Response: We thank the reviewer for this helpful suggestion and added the number of eyes per group to the figure legends for each analysis method.

Page 6 Line 9: This is a very interesting finding, why is this not shown?

Response: As suggested by the reviewer, we prepared a figure of the not shown data and added this one to figure 3. Here, the relative *LAMP1* mRNA expression was investigated via qRT-PCR. The expression level of the anti-VEGF treated groups were compared to the one of the ischemic group. A slight trend to a lower *LAMP1* mRNA expression was detected in retinae treated with ranibizumab (p=0.13; Figure 3G).

Discussion:

First lines: Again, state here that there the results to ischemia were not significant.

Response: We revised the discussion part concerning this aspect. We mentioned at the beginning of the discussion that “there were no significant differences between the untreated ischemic and the anti-VEGF treated groups”.

Page 10, Line 19+20. Please elaborate more on the toxicity of VEGF found in 33.

Response: As suggested by the reviewer, we provided more information about the investigation of the toxicity of VEGF inhibitors which were performed by Thaler et al. (Thaler et al., 2010; *Toxicity testing of the vegf inhibitors bevacizumab, ranibizumab and pegaptanib in rats both with and without prior retinal ganglion cell damage.* Acta Ophthalmol, 88, e170-176). We added the following sentences to the discussion: “Thaler et al. performed electron microscopy and evaluated retrogradely labelled RGCs after intravitreal injection of bevacizumab and ranibizumab in healthy rats and those with NMDA-induced RGC damage. Neither in healthy animals, nor in NMDA treated animals, any differences in structure, morphology or the number of RGCs after anti-VEGF treatment were found in comparison to the respective control groups.”

The authors need to discuss the results of their similar study published in PLosONE: Why did they see significant results in that study but not in this one. Was it because in the first study Ranibizumab was injected after three days? Maybe also the sample size number was too low in this study as there was more comparison due to the testing of Bevacizumab. Maybe there are significant differences but a higher sample size is needed?

Response: We thank the reviewer for this advice. We agree with the reviewer, that in the mentioned previous manuscript our group could show a significant protection of RGCs by ranibizumab treatment, while in the current study just a trend was seen concerning RGC protection. A possible explanation could be the analyzed number of eyes per group, which was lower in the current study (n=5-6/group vs. n=7-10/group). Nevertheless, the focus of our study was the comparison of the two different VEGF inhibitors and we were also able to show a certain protective effect on this cell type after ranibizumab therapy.

M&M:

The standard human dosing for anti-vegf injections is 0.5 mg in 50µl for ranibizumab and 1.25 mg in 50µl for bevacizumab. The authors used 0.5mg in 5µl for both drugs. First, how did the authors concentrate ranibizumab to the higher concentration? Second, why did the use a lower concentration for bevacizumab than the clinical dose? Maybe the clinical dose would have caused better effects. It does not make sense to use the same concentration for both drugs, as bevacizumab is a full antibody and therefore needs to be higher concentrated than the truncated ranibizumab to have the same amount of “efficient parts.”

Response: We agree with the reviewer, that the administered dose in humans is different for the two VEGF inhibitors and that the efficacy of these is different. However, we wanted to draw a comparison at the same dose since we have seen positive and protective effects of ranibizumab in previous studies of our group (Joachim et al., 2017) by applying 0.05 mg. Therefore, we wanted to keep this dose also for the comparative study. This allows to make a possible comparison regarding the beginning of therapy. In addition, we consulted previous publications that also used this dose for both VEGF inhibitors. Especially, for bevacizumab corresponding effects could be determined (Abcouwer et al., 2010; *Effects of ischemic preconditioning and bevacizumab on apoptosis and vascular permeability following retinal ischemia-reperfusion injury.* Invest Ophthalmol Vis Sci; 51(11): 5920-33). As the volume of rat vitreous varies from 12 µl to 56 µl and the human vitreous volume comprises about 4 ml, Lu and Adelman calculated for administration of the VEGF inhibitors in rats an equivalent dose to human dose (1/30 – 1/170 of humans) (Lu and Adelman, 2009; *Are intravitreal bevacizumab and ranibizumab effective in a rat model of choroidal neovascularization.* Graefes Arch Clin Exp Ophthalmol; 247: 171-177). Equally, we performed a calculation for an equivalent dose for the administration including an adaption of the volume and applied 0.05 mg in 5 µl. We did not concentrate ranibizumab to a higher concentration.

Table 1:

Primer efficiency is rather low for Bad and Brn3a. The authors should redo those experiments with a more efficient primer pair.

Response: As suggested by the reviewer, the qRT-PCR analyses for relative *Bad* and *Brn-3a* expression were repeated by using new primer pairs with a better primer efficiency (both: 1.0). The corresponding figures were exchanged (figure 2D: *Brn-3a*; figure 2E: *Bad*).

5´and 3´should be given.

Response: We thank the reviewer for this advice and added the missing direction for the primer sequences to table 2.

Conclusion:

Should be revised and be more modest.

Response: As suggested by the reviewer, we revised the conclusion. We now point out that the comparison of the two inhibitors was the focus of the investigations.
