# Supplementary material for: Fewer Functional Deficits and Reduced Cell Death after Ranibizumab Treatment in a Retinal Ischemia Model
Source: Int J Mol Sci. 2018 May 31;19(6):1636. doi: 10.3390/ijms19061636 (PMC6032266; doi:10.3390/ijms19061636)
Supplement: Supplementary file 1 [file ijms-19-01636-s001.zip › Supplement+ReviewerComments/Revision_Supplement_RanibizumabTreatmentRetinalIschemia_MPalmhofSJoachim.docx]

**Supplement Table 1:** **A.** ERG measurements of all groups, control, ischemic, and anti-VEGF treated ones (beva, rani), were recorded at 0.1, 0.3, 1, 3, 10, and 25 cd/m^2^. The values (mean±SEM) for the a-wave amplitude are shown. A significant, light intensity dependent decrease of the amplitude could be noted in all ischemic groups, the untreated and the anti-VEGF treated ones (p<0.05) in comparison to control eyes. The ranibizumab treated eyes only showed a significantly reduced amplitude at 0.3 cd/m^2^ (p=0.03). **B.** The values (mean±SEM) for the b-wave amplitude and the corresponding light intensities (0.1, 0.3, 1, 3, 10, and 25 cd/m^2^) of the ERG measurement are listed. All ischemic groups, the untreated and both anti-VEGF treated ones, displayed a significant decrease of the b-wave amplitude in comparison to control eyes (p<0.001). The ranibizumab treated group showed a less reduced b-wave amplitude, especially at higher light intensities. At the light intensity of 3 cd/m^2^ a significant amplitude increase was observed in comparison to the ischemic group (p=0.03).

**Supplement Figure 1:** **A.** Exemplary retinal cross-sections of the retina. Sections of all four groups, control, ischemia, bevacizumab, and ranibizumab treated ones, were stained for RGCs with anti-Brn-3a (green), for apoptotic cells with anti-Bax (red) and for cell nuclei using DAPI (blue). In all ischemic groups fewer Brn-3a^+^ RGCs and more Bax^+^ apoptotic cells were detected. These were localized in the GCL. **B.** Anti-LC3BII was used to label early autophagy (green), while the late autophagy was visualized with anti-LAMP1 (red). Cell nuclei were marked with DAPI (blue). In general, positive autophagy signals were mainly observed in the GCL. More autophagocytotic cells were noted in ischemic retinae. **C.** An anti-Iba1 antibody was used to stain microglia (green), while activated microglia were labelled with anti-ED1 (red). Microglia as well as their activated type were noted in the GCL, IPL, and INL. In comparison to the control group, more Iba1^+^ and ED1^+^ microglia could be observed after ischemic injury. **D.** AII amacrine cells were visualized with anti-parvalbumin (green) and cholinergic amacrine cells with anti-ChAT (green), while anti-recoverin was used to label cone bipolar cells (red). DAPI was used to mark cell nuclei (blue). Cell bodies of AII and cholinergic amacrine cells as well as cone bipolar cells were present in the INL. Furthermore, some displaced cholinergic amacrine cells were visible in the GCL. After ischemic injury, fewer amacrine cells as well as cone bipolar cells were seen. In addition, a distinct stratification in the IPL could only be noted in the control group concerning ChAT immunoreactivity. This was also present in an attenuated form in ranibizumab treated retinae. After treatment with bevacizumab and ranibizumab, the loss of bipolar cells seemed to be less prominent. Abbreviations: GCL: ganglion cell layer, IPL: inner plexiform layer, INL: inner nuclear layer, OPL: outer plexiform layer, ONL: outer nuclear layer, PR: photoreceptors, Arrows: colocalization. Scale bar: 20 µm.

**Supplement Figure 1**
